# Characterizing the cytotoxic effects and several antimicrobial phytocompounds of *Argemone mexicana*

**DOI:** 10.1371/journal.pone.0249704

**Published:** 2021-04-07

**Authors:** Danielle Annette Orozco-Nunnelly, Jeffery Pruet, Clara Patricia Rios-Ibarra, Estefany Lucia Bocangel Gamarra, Theodore Lefeber, Teodora Najdeska

**Affiliations:** 1 Department of Biology, Valparaiso University, Valparaiso, IN, United States of America; 2 Department of Chemistry, Valparaiso University, Valparaiso, IN, United States of America; 3 Department of Biotechnology, Tecnológico de Monterrey, Campus Guadalajara, Guadalajara, Jalisco, México; 4 Institute of Research in Biomedical Sciences, University Center for Health Science, University of Guadalajara, Guadalajara, Jalisco, México; Cairo University, EGYPT

## Abstract

Commonly called the Mexican prickly poppy, *Argemone mexicana* is a stress-resistant member of the Papaveraceae family of plants that has been used in traditional medicine for centuries by indigenous communities in Mexico and Western parts of the United States. This plant has been exploited to treat a wide variety of ailments, with reported antimicrobial and antioxidant properties, as well as cytotoxic effects against some human cancer cell lines. Due to its various therapeutic uses and its abundance of secondary metabolites, *A*. *mexicana* has great potential as a drug discovery candidate. Herein, the germination conditions of *A*. *mexicana* are described and the cytotoxic activities of different parts (seeds, leaves, inner *vs*. outer roots) of the plant from methanol or hexane extracts are preliminarily characterized against cells of seven unique organisms. When comparing 1 mg of each sample normalized to background solvent alone, *A*. *mexicana* methanol outer root and leaf extracts possessed the strongest antimicrobial activity, with greatest effects against the Gram-positive bacteria tested, and less activity against the Gram-negative bacteria and fungi tested. Additionally, using the MTT colorimetric assay, the outer root methanol and seed hexane extracts displayed pronounced inhibitory effects against human colon cancer cells. Quantification of c-MYC (oncogene) and APC (tumor suppressor) mRNA levels help elucidate how the *A*. *mexicana* root methanol extract may be affecting colon cancer cells. After ultra-performance liquid chromatography coupled with mass spectrometry and subsequent nuclear magnetic resonance analysis of the root and leaf methanol fractions, two main antibacterial compounds, chelerythrine and berberine, have been identified. The roots were found to possess both phytocompounds, while the leaf lacked chelerythrine. These data highlight the importance of plants as an invaluable pharmaceutical resource at a time when antimicrobial and anticancer drug discovery has plateaued.

## Introduction

According to the World Health Organization, infectious diseases (lower respiratory illnesses, diarrheal diseases, tuberculosis) and cancers of the lower respiratory system account for four of the top ten global causes of death [1]. When looking at low-income countries, the frequency of communicable diseases increases to seven of the top ten leading causes of death, and for high-income countries, cancer becomes overrepresented and includes colon, rectum and breast cancers [1]. Traditional medications to treat such diseases include antimicrobial drugs for infectious illnesses and chemotherapy or targeted drug therapy for cancers. While there was a surge in antimicrobial and anticancer drug discovery in the mid-twentieth century, more recently, the development of such drugs has sharply declined. This problem is exemplified with antibiotic medications, where nine new classes of drugs were deployed between 1940 and 1960; while from 1970–2005, only two new classes of antibiotics were released [2]. Such information is concerning given the growing number of drug-resistant microorganisms and the rise of difficult-to-treat ‘superbugs,’ such the nosocomial pathogen MRSA (methicillin resistant *Staphylococcus aureus*) [3] and the recently emergent antibiotic-resistant ST131-H30 *E*. *coli* strain [4], to name just a few. In 2014, global deaths due antimicrobial resistance were approximately 700,000 per year, but due to the increasing rate of superbug creation, this number has been predicted to jump to 10 million per year by 2050, at a cost of $100 trillion USD [5]. Thus, the need for new antimicrobial and anticancer treatments is at the forefront of modern healthcare today.

Plants naturally produce a robust supply of novel metabolic compounds that have provided an invaluable drug discovery resource for centuries. From 1981 to 2010, it is estimated that nearly 50% of all cancer drugs originated from natural products [6], many of which were derived from terrestrial plants [7]. Likewise, plants produce a host of antimicrobial agents, including a wide variety of natural defense compounds, such as phenolics, terpenoids, alkaloids, polyacetylenes, lectins and polypeptides [8]. However, with the advent of modern antibiotic drugs mainly of bacterial, fungal and synthetic sources, many of these natural plant-derived antibiotic compounds have been left unexplored.

*Argemone mexicana*, a stress-resistant member of the Papaveraceae family of plants, has been reported to possess a wide-range of biological activities, such as antibacterial [9–12], antifungal [13–16], antiviral [17–19], anthelmintic [20–22], antioxidant [23–25] and cytotoxic/anticancer [15, 26, 27] actions. This plant is native to the West Indies, but today it can also be found in Mexico and throughout the Americas [28]. It has been used in traditional medicine for centuries; for example, Native Americans (since the time of the Aztecs), have used various parts of the plant as an analgesic tea to relieve kidney pain, for migraines, as well as during and after childbirth [29]. Due to its strong medicinal potential, *A*. *mexicana* has been analyzed by several research groups to determine its main secondary metabolites, which include phenolics (such as flavonoids and tannins), terpenoids (such as glycosides) and N-containing compounds (such as alkaloids), as well as saponins and steroids [30, 31]. Although some studies have been conducted to better understand these compounds and the biological activities of *A*. *mexicana* (reviewed in [31, 32]), the phytochemicals possessing many of these biological activities have not yet been identified. Thus, this plant possesses great potential for the discovery of novel antibiotic and anticancer compounds.

In the work herein, a comprehensive evaluation of the cytotoxic effects of the *A*. *mexicana* plant is provided. Both methanol and hexane extracts of four plant parts (leaves, seeds, inner *vs*. outer roots) were tested against the cells of seven unique species: four bacteria, two fungi and a human colon cancer cell line that had not previously been assessed against *A*. *mexicana* in the literature. Guided by these results and employing analytical chemistry techniques, two main antimicrobial compounds of the plant, berberine and chelerythrine, were separated and identified. Likewise, *A*. *mexicana* germination conditions were evaluated, as well as the antibiotic production potential at two root developmental states. Utilizing qPCR, the previously unexplored effects that the root methanol extract has on two colon cancer genes were explored. The data considered expand upon previous studies into the antibiotic and cytotoxic potential of the *A*. *mexicana* plant and provide novel information concerning the impact of this plant on human colon cancer cells and regarding the presence and preferential distribution of the berberine and chelerythrine phytocompounds in the roots and/or leaves of *A*. *mexicana*.

## Materials and methods

### Plant materials

With the permission of the landowner, *A*. *mexicana* plants were identified and harvested from an abandoned sugarcane field on private land in Kahului, Hawaii by Rick Sheffield of Sheffield Seed Company, Inc. After removing soil, plants were separated into leaves, seeds and roots. All plant material was then allowed to dry in paper bags at 22°C and stored until further use. Plant voucher material will be deposited into the herbarium at the Field Museum of Natural History in Chicago, IL.

### Gibberellic acid germination experiments

Two *A*. *mexicana* seeds were planted per soil pod and watered with equal amounts of either 100 mg/L or 1000 mg/L gibberellic acid (GA; C_19_H_22_O_6_) (Fisher Scientific, Pittsburgh, PA, USA) solutions or with a control solution (containing no GA). Ten soil pods were used per group. Ethanol (Fisher Scientific, Pittsburgh, PA, USA), the solvent used to prepare the stock GA solution, was used in place of GA in the 100 mg/L GA and control solutions. Seedlings were kept in small greenhouses under a 16/8 light cycle and mean germination rate per pod was calculated after 30 days.

### Extraction procedure

Whole *A*. *mexicana* plants were separated into seeds, leaves or roots and allowed to dry in paper bags at 22°C. Inner *vs*. outer root was separated using a fine blade only after plants were completely dry. 2 g of each sample was then homogenized using a mortar and pestle. The dried, homogenized samples were extracted using the following method: The powdered sample was macerated in either methanol or hexane (Fisher Scientific, Pittsburgh, PA, USA) using a 1:4 (plant material:solvent) ratio at 200 rpm, 35°C for 72 h. The mixture was centrifuged at 5,000 x g for 5 min, and the supernatant was filtered through a 0.2 μM PTFE membrane (Fisher Scientific, Pittsburgh, PA, USA). The solvent was fully evaporated at 35°C in a fume hood and the remaining dehydrated filtrate was quantified and tested for bioactivity.

### Antimicrobial disc diffusion assay

Blank antibiotic sensitivity discs (Fisher Scientific, Pittsburgh, PA, USA) were impregnated with 1 mg of *A*. *mexicana* extract from either the seed, leaf, inner root or outer root. Once the solvent evaporated, discs were placed onto a media plate with a lawn of one of the following microorganisms: *Staphylococcus aureus*, *Bacillus cereus*, *Escherichia coli*, *Proteus mirabilis*, *Candida albicans* or *Saccharomyces cerevisiae* (Carolina Biological Supply Company, Burlington, NC, USA). After 48 h of growth, zones of inhibition were measured using a ruler for each disc and mean zones of inhibition in millimeters were calculated. The antibiotics vancomycin, streptomycin and/or fluconazole were used as positive controls. The solvents (methanol or hexane alone) were used as negative controls and showed no zones of inhibition.

### Cell culture

Human T84 colon cancer cells were a generous gift from Patrice Bouyer at Valparaiso University, originally described in [33], and human RKO colon cancer cells were obtained from ATCC (American Type Culture Collection, Manassas, VA, USA). All cell lines were maintained and cultured at 37°C with 5% CO_2_, as detailed in [34], using advanced Dulbecco’s modified Eagle’s medium (ADMEM) (GIBCO-BRL, Grand Island, NY, USA) supplemented with 2% fetal bovine serum (FBS), 1% penicillin/streptomycin mixture, 1% glutamine and 1% essential amino acids (all obtained from Fisher Scientific, Pittsburgh, PA, USA). Cells were washed with 5 mL of sterile 1X PBS (Fisher Scientific, Pittsburgh, PA, USA) for a 75 cm^2^ bottle or with 2 mL for a 25 cm^2^ bottle. To detach the monolayer of cells from the surface, 1 mL of trypsin (Fisher Scientific, Pittsburgh, PA, USA) was added and incubated at 37°C for 5–10 min; to neutralize the trypsin, an equal volume of culture media was added to the trypsin/cell mixture after transfer to a 15 mL falcon tube. Cells were centrifuged at 1,000 rpm for 5 min at room temperature; then the supernatant (media-trypsin) was then removed, and 2–4 mL of culture media was added to the pellet, which was carefully mixed to resuspend. Next, a previously calculated volume of cells was added to a 75 cm^2^ bottle with 10 mL of culture media or to a 25 cm^2^ bottle with 5 mL of culture media. Cells were incubated at 37°C with 5% CO_2_ until they reached 90% confluency.

### Extract treatments and viability assay of colon cancer cells

In 24-well plates, cells were seeded at a density of 30,000 cells per well for T84 cells or at a density of 10,000 cells per well for RKO cells. To compare the effects of different extracts, T84 cells were treated for 1 h with 1 mg of dehydrated extract into 500 mL culture media, where all solvent was allowed to fully evaporate before bringing the dehydrated extract back up into culture media. Cells were then subjected to the Vybrant® MTT Cell Proliferation Assay Kit (Molecular Probes, Eugene, OR, USA) to assess cell metabolic activity. Results were quantified using a plate reader at 570 nm, and the mean percentage of viable cells normalized to the control (evaporated methanol or hexane alone) was calculated. To determine the ideal root methanol concentration with which to treat cells without killing them (for RNA harvest and subsequent qPCR analysis), RKO cells were treated with 0.0625 μg/μL, 0.125 μg/μL, 0.25 μg/μL, 0.50 μg/μL or 1.0 μg/μL dehydrated root methanol extract or no treatment (evaporated methanol alone). Again, all solvent was allowed to fully evaporate before bringing the dehydrated extract back up into culture media. Cytotoxic effects were assessed via the MTT assay as described above for cell viability after 72 h.

### Total RNA isolation

Total RNA was extracted from RKO cells 24, 48 and 74 h after treatment with 0.0625 μg/μL root methanol extract or methanol alone using TRIzol (Invitrogen, Waltham, MA, USA) according to the manufacturer’s instructions. Briefly, the contents of each well were mixed with TRIzol and transferred to a 1.5 mL Eppendorf tube. This mixture was incubated for 5 min at room temperature to allow complete dissociation of nucleoprotein complexes. 40 μL of chloroform (Sigma-Aldrich, St. Louis, MO, USA) per every 200 μL of Trizol was then added and mixed by inversion for 15 seconds; the tube was incubated on ice and centrifuged for 15 min at 13,000 rpm at 4°C. The resulting aqueous phase was recovered, added to 100 μL isopropanol (Sigma-Aldrich, St. Louis, MO, USA) and incubated at -80°C for 1 h or overnight. After incubation, this mixture was centrifuged at 13,000 rpm for 15 min at 4°C. Subsequently, the supernatant was removed, and the pellet washed with 200 μL of 70% ethanol (prepared with DEPC water), then centrifuged at 13,000 rpm for 5 min at 4°C. Finally, the resulting ethanol layer was carefully removed using a micropipette, and the pellet was re-suspended in 20 μL of water with 0.3 μL RNaseOUT (Fisher Scientific, Pittsburgh, PA, USA). The total RNA samples were stored at -80°C until further use.

### Reverse Transcription (RT)

“Mix 1” was prepared with by combining 5 μL (~1000 ng) of isolated RNA, 5.5 μL of DEPC H_2_O and 1 μL of ‘Random’ primers (Invitrogen, Waltham, MA, USA) and was incubated at 72°C for 10 min in a thermocycler, followed by ice for 3 min. Simultaneously, “Mix 2” was prepared by adding the following components: 2 μL of 0.1 M DTT (Sigma-Aldrich, St. Louis, MO, USA), 0.5 μL of 40 U/μL RNaseOUT (Fisher Scientific, Pittsburgh, PA, USA), 1 μL of dNTP’s (Invitrogen, Waltham, MA, USA), and 1 μL of 200 U/μL M-MLV Reverse Transcriptase (Invitrogen, Waltham, MA, USA) into 4 μL of 5X RT Buffer. 8.5 μL of “Mix 1” was then added to the entire volume of “Mix 2” and incubated under the following series of conditions: 10 min at 25°C, then 1 h at 37°C, then 5 min at 94°C, then 30 min at 4°C. The resulting cDNA was stored at -20°C until use.

### Real time PCR (qPCR)

The reaction components (in a final volume of 20 μL) included: 10 μL of 2X SybrGreen (Invitrogen, Waltham, MA, USA), 7 μL of DEPC water, 1 μL of the forward primer (40 mM APC, c-MYC or Actin), 1 μL of the reverse primer (40 mM APC, c-MYC or Actin), and 1 μL of 10 ng/μL cDNA. The following reaction conditions were used: 2 min at 50°C, 10 min at 95°C, then 45 cycles of 15 seconds at 95°C and 1 min at 60°C. The exact primers used (Invitrogen, Waltham, MA, USA) are listed below:

**Table:** 

APC FW	5´-TTATGGAAGCCGGGAAGGA-3´
APC RV	5´-TGGAAATGAACCCATAGGAACAG-3´
c-MYC FW	5′-TCAAGAGGCGAACACACAAC-3′
c-MYC RV	5′-GGCCTTTTCATTGTTTTCCA-3′
Actin FW	5´-TGGACTTCGAGCAAGAGATGG-3´
Actin RV	5´-GGAAGGAAGGCTGGAAGAGTG-3´

### Analysis and purification of crude root and leaf extracts

All crude methanol root and leaf extracts were analyzed by TLC (thin layer chromatography), using precoated glass-backed UV254 silica plates (Analtech, Newark, DE, USA), developed using 9:1 DCM:MeOH (0.2% NH_3_). All spots were visualized with a UV lamp, at both 254 nm and 365 nm. The TLC plates were furthered visualized with an iodine stain. The methanol solution of the crude root or leaf extract was suspended onto a 10:1 silica-to-sample mass ratio. This slurry was concentrated under reduced pressure to provide a dry silica plug. The silica was then packed into an appropriately sized empty RediSep Rf cartage (Teledyne Isco, Lincoln, NE, USA) for purification on a CombiFlash Rf+ automated chromatography system (Teledyne Isco, Lincoln, NE, USA). Pre-packed RediSep Rf High-Performance GOLD silica columns (Teledyne Isco, Lincoln, NE, USA) were used for all separations. The solvent gradient was varied from 100% DCM to 70% DCM and 30% MeOH (with 1% NH_3_) over a 30–35 min period with a 20 mL/min flow rate. Peaks were detected using wavelengths set at λ1 = 254 nm and λ2 = 340 nm with a 5x signal gain. Fractions were pooled based on their similar elution times and absorbances and labeled as A-thru-F (for the root methanol fractions) or 1-thru-10 (for the leaf methanol fractions), with further subfractionation performed on specific samples. The purity of the column fractions were initially analyzed by TLC, using precoated glass-backed UV254 silica plates (Analtech, Newark, DE, USA), developed in 9:1 DCM:MeOH (with 0.2% NH_3_) and monitored at wavelengths of 254 nm and 365 nm. Purity was furthered evaluated using an iodine stain. Additional analyses related to purity were conducted using ultra-performance liquid chromatography (UPLC) as described below. Spectroscopically pure samples were recovered by use of 1000μm thickness 20x20cm preparative silica TLC plates (Analtech, Newark, DE, USA) to first separate the crude methanol extract. Bands corresponding to the components of interest were then cut from the plate and this silica plug was further purified on the CombiFlash Rf+ automated chromatography system (Teledyne Isco, Lincoln, NE, USA) as described above.

### Mass spectrometry analysis

Active components separated as described above were analyzed by ultra-performance liquid chromatography coupled with mass spectrometry using a Waters^TM^ Acquity UPLC H-class system equipped with a QDa detector (Waters Corporation, Milford, MA, USA). Chromatographic separations were performed with a Waters^TM^ Acquity UPLC BEH C18 column (1.7 μm, 2.1 × 50 mm) and a gradient flow beginning with 10% acetonitrile (0.1% formic acid) and 90% water (0.1% formic acid), changing to 80% acetonitrile and 20% water at 6 min. The solvent returned to the initial formulation at 12 min. The solvent flow rate was 0.23 mL min^−1^, with the column and sample compartment temperatures held constant at 20°C. Mass spectrometry was utilized in positive scan mode, with a cone voltage of 15 V and a capillary voltage of 1.5 kV. High-resolution mass spectrometry data was collected on a Bruker micrOTOF II (Bruker Scientific, Billerica, MA, USA) in positive scan mode. Since the two main phytochemicals later identified were found to exist as fixed-charged species, these appeared as their M+ ions, but their neutral solvent adducts appeared as the M+H ions. After subsequent identification of the phytochemicals, analytical standards were purchased (Sigma-Aldrich, St. Louis, MO, USA) and used to validate and confirm uniformity in the method results.

### Nuclear Magnetic Resonance (NMR) analysis

All ^1^H-NMR were recorded in chloroform-*d* (CDCl_3_; Sigma-Aldrich, St. Louis, MO, USA) on a 400 MHz Bruker spectrometer using the solvent as the reference. Chemical shifts are given in parts per million (ppm). The ^13^C-NMR was recorded in either chloroform-*d* or methanol-*d*_4_ (CD_3_OD; Sigma-Aldrich, St. Louis, MO, USA), depending on the solubility of the sample.

### Statistical analysis

Data shown in figures were entered into Microsoft ® Excel for Mac Version 16.44 (Microsoft Corporation, Albuquerque, NM, USA), where mean and Standard Error of the Mean (SEM) are shown for most graphic depictions. For gibberellic acid germination experiments, mean germination rate per pod (with ten pods per group) is displayed with SEM, and a two-tailed *T*-test was used with significance set at *P* ≤ 0.05. For the antimicrobial disc diffusion assay, mean zones of inhibition in millimeters for five biological replicates are presented with associated SEM. For T84 and RKO human colon cancer toxicity experiments, mean percentage of viable cells normalized to the control (solvent alone) for three biological replicates is shown with associated SEM. Real-time PCR results are displayed as mean transcript level of three biological replicates shown with associated SEM and normalized to Actin mRNA levels. Statistical significance among transcript means was determined through a one-way analysis of variance (ANOVA) with SPSS for Windows, v.21 (IBM Corp., Armonk, NY, USA), where differences were considered statistically significant with a value of *P* ≤ 0.05.

## Results and discussion

**Fig 1:**
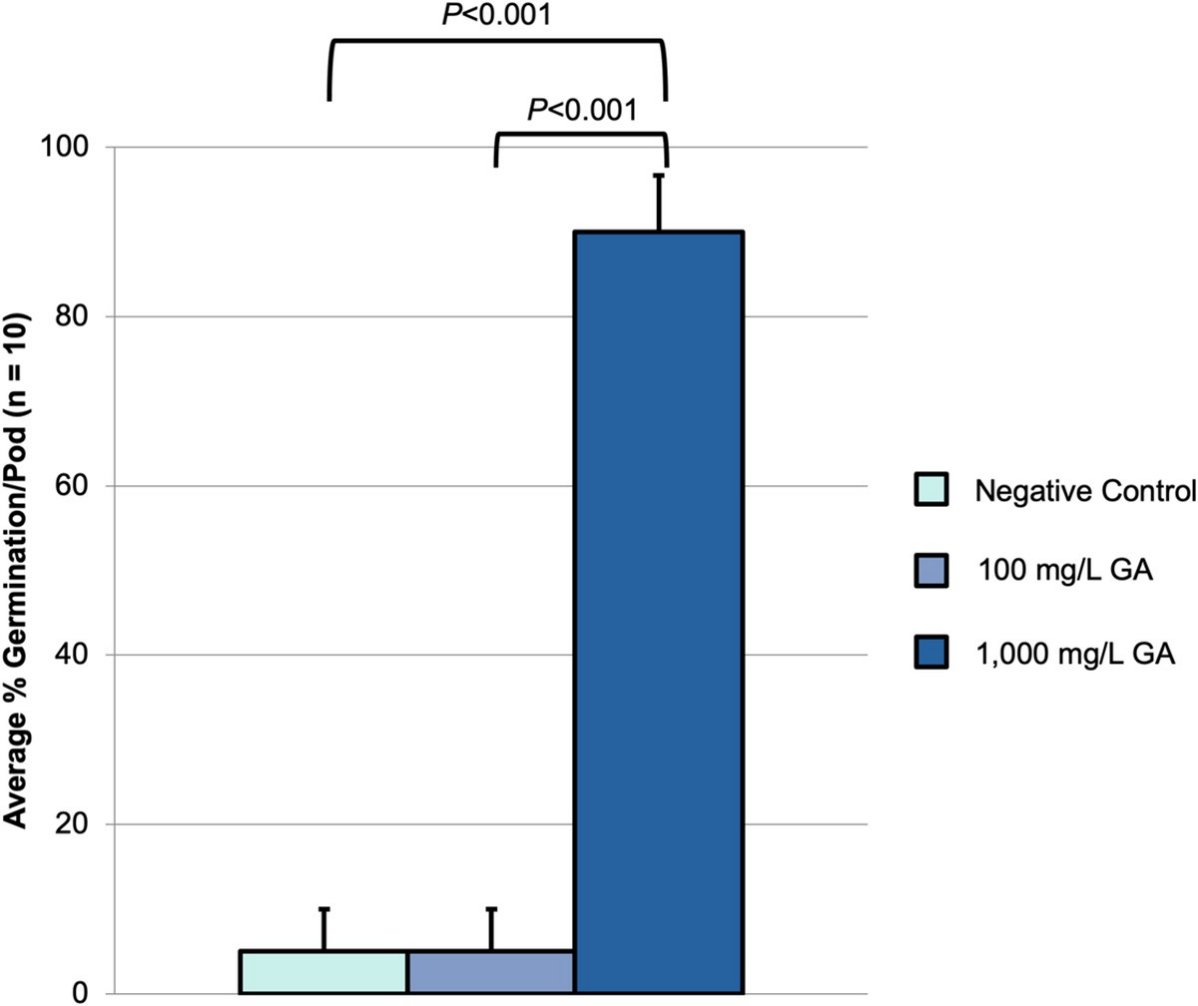
Gibberellic acid germination experiments. Two *A*. *mexicana* seeds were planted per soil pod and watered with equal amounts of either the negative control (no GA), 100 mg/L or 1000 mg/L gibberellic acid solutions, with 10 total pods used per group. Ethanol (the solvent used to prepare the stock GA solution) was used in place of GA in the negative control and 100 mg/L GA water solutions. Seedlings were kept in small greenhouses under a 16/8 light cycle and scored after 30 days. n = 10 pods, with mean germination rate per pod displayed with associated SEM. A two-tailed *T*-test was used to determine statistically significant differences between means, with significance set at *P* ≤ 0.05.

*A*. *mexicana* seeds (originally obtained from Sheffield Seed Company, Inc.) displayed low germination rates. In an attempt to increase the percentage of germination for stock seed maintenance, seeds were treated with solutions of either 100 mg/L or 1,000 mg/L of the phytohormone gibberellic acid (GA) or with a control solution (lacking GA). Ethanol, the solvent used to prepare the stock GA solution, was used in place of GA in the 100 mg/L GA and control solutions. Gibberellic acid is thought to play a role in breaking seed dormancy and initiating germination [35]. After 30 days, germination rates were compared and quantification of the mean percent germination indicated significant differences between the negative control (no GA) *vs*. 1,000 mg/L GA (*P*<0.001, two-tailed *T*-test) and between 100 mg/L *vs*. 1,000 mg/L GA (*P*<0.001, two-tailed *T*-test), where the negative control (no GA), 100 mg/L and 1,000 mg/L treatments exhibited means of 5%, 5% and 90%, respectively (**Fig 1**).

**Fig 2:**
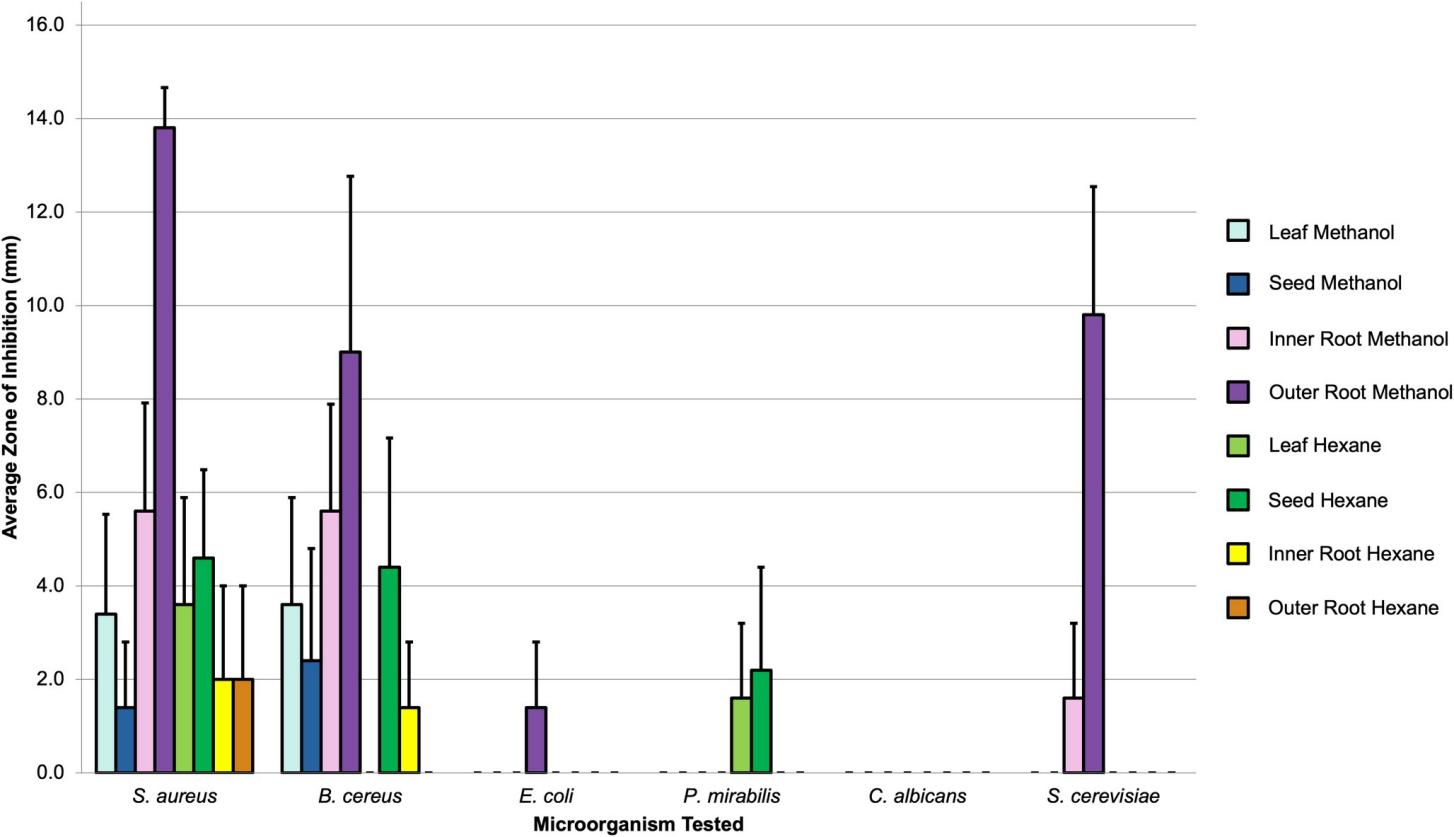
Antimicrobial disc diffusion assay. 1 mg of either methanol or hexane extract for leaf, seed, inner root or outer root was plated against six different microorganisms (*S*. *aureus*, *B*. *cereus*, *E*. *coli*, *P*. *mirabilis*, *C*. *albicans* or *S*. *cerevisiae*). The mean zone of inhibition in millimeters for five biological replicates is shown with associated SEM. Vancomycin, streptomycin and/or fluconazole were used as positive controls, and solvents alone were used as negative controls.

Initially, portions of the *A*. *mexicana* plant (seeds, leaves, inner roots and outer roots) were extracted via maceration with solvents of various polarities. Preliminary results indicate that outer root methanol extracts possess the greatest antimicrobial activity, with largest effects against the Gram-positive bacteria tested (*S*. *aureus* and *B*. *cereus*) for nearly all extracts (**Fig 2**). This result is consistent with several other studies that have found antibacterial activity in the crude methanol and hexane extracts of various parts of the *A*. *mexicana* plant, such as from stem hexane extracts [11], leaf methanol extracts [12, 36] and seed methanol extracts [37]. Interestingly, except for one condition (outer root methanol against *S*. *cerevisiae*), all treatments had little to no effect against either the Gram-negative bacteria (*E*. *coli* and *P*. *mirabilis*) or fungi (*C*. *albicans* and S. *cerevisiae*) that were tested. Although it is common for Gram-negative diderm bacteria to be less sensitive than Gram-positive organisms, this finding is contrary to what has been reported of the crude fruit methanol extract, which was shown to be more effective against Gram-negative than Gram-positive bacteria [38]. This suggests that the main antimicrobial compounds in the leaves, seeds and roots may be targeting a unique feature of Gram-positive bacteria, such as the peptidoglycan cell wall. In an effort to increase the yield of antimicrobial compounds in the extracts, use of the Soxhlet extraction technique was also explored. However, this was found to show no significant enhancement in activity or yield (**S1 Fig**); therefore, the maceration technique was used exclusively for this work.

**Fig 3:**
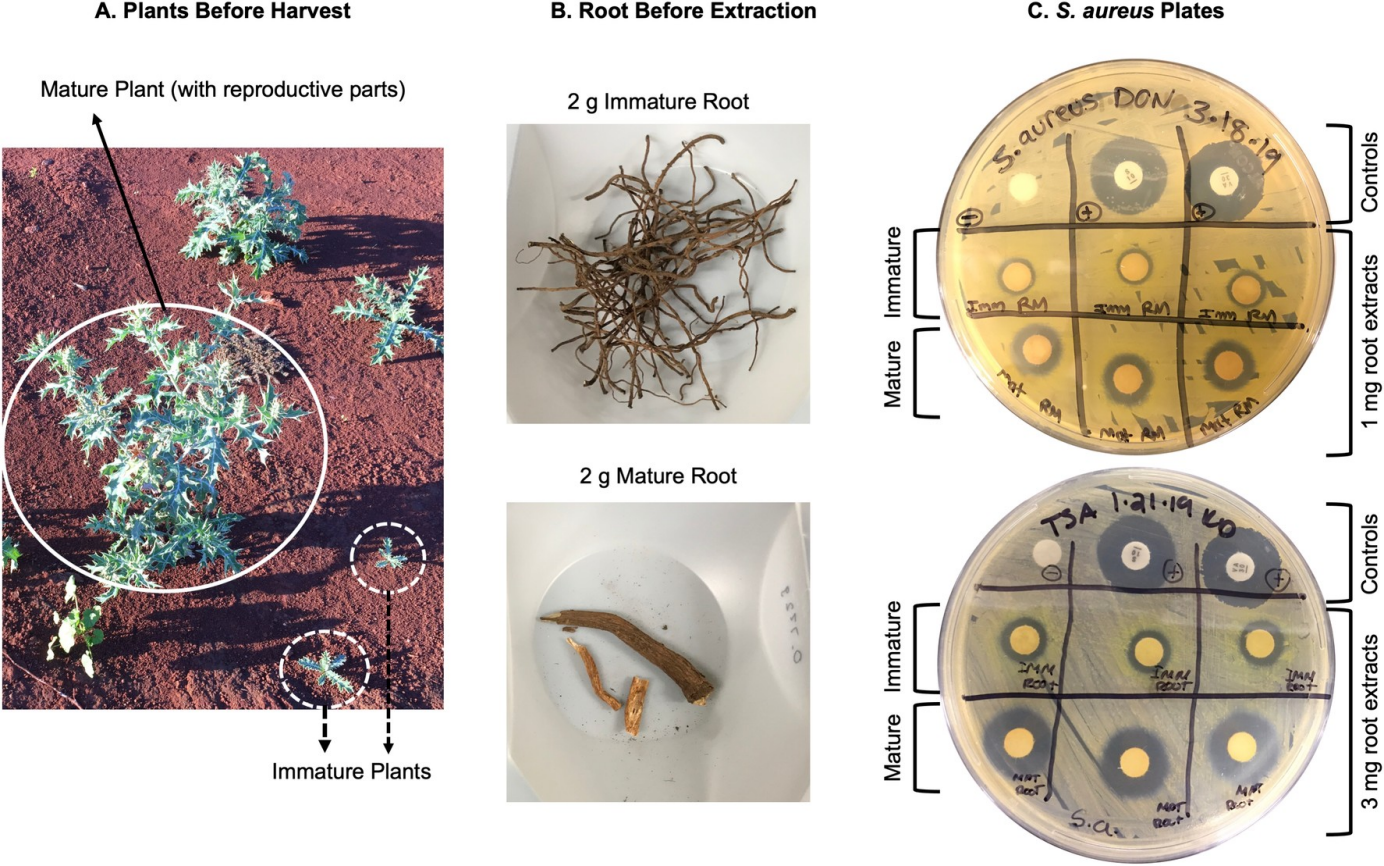
Antimicrobial effect comparison of immature *vs*. mature root. Immature *vs*. mature roots were harvested from *A*. *mexicana* plants either without or with reproductive structures (panel A, photo taken by Sheffield Seed Company, Inc.). Equal amounts of either immature or mature root (pictured in panel B) was then extracted in methanol, and either 1 mg (panel C, top) or 3 mg (panel C, bottom) of the extract was plated against *S*. *aureus*, using streptomycin and vancomycin as positive controls and methanol alone as the negative control.

As young seedlings are establishing themselves and building their arsenal of defense compounds, they are often more susceptible to death by both abiotic and biotic factors [39]. To better understand the role that developmental state may have on the production of antibacterial compounds (established in **Fig 2**), mature *vs*. immature roots were examined for inhibitory activity against *S*. *aureus* (**Fig 3**). The methanol extracts from the mature roots were found to have greater antibacterial activity when compared to the immature extracts (**Fig 3C**), suggesting that these antimicrobial compounds are produced in greater amounts after the plant has transitioned from a vegetative to a reproductive mode.

**Fig 4:**
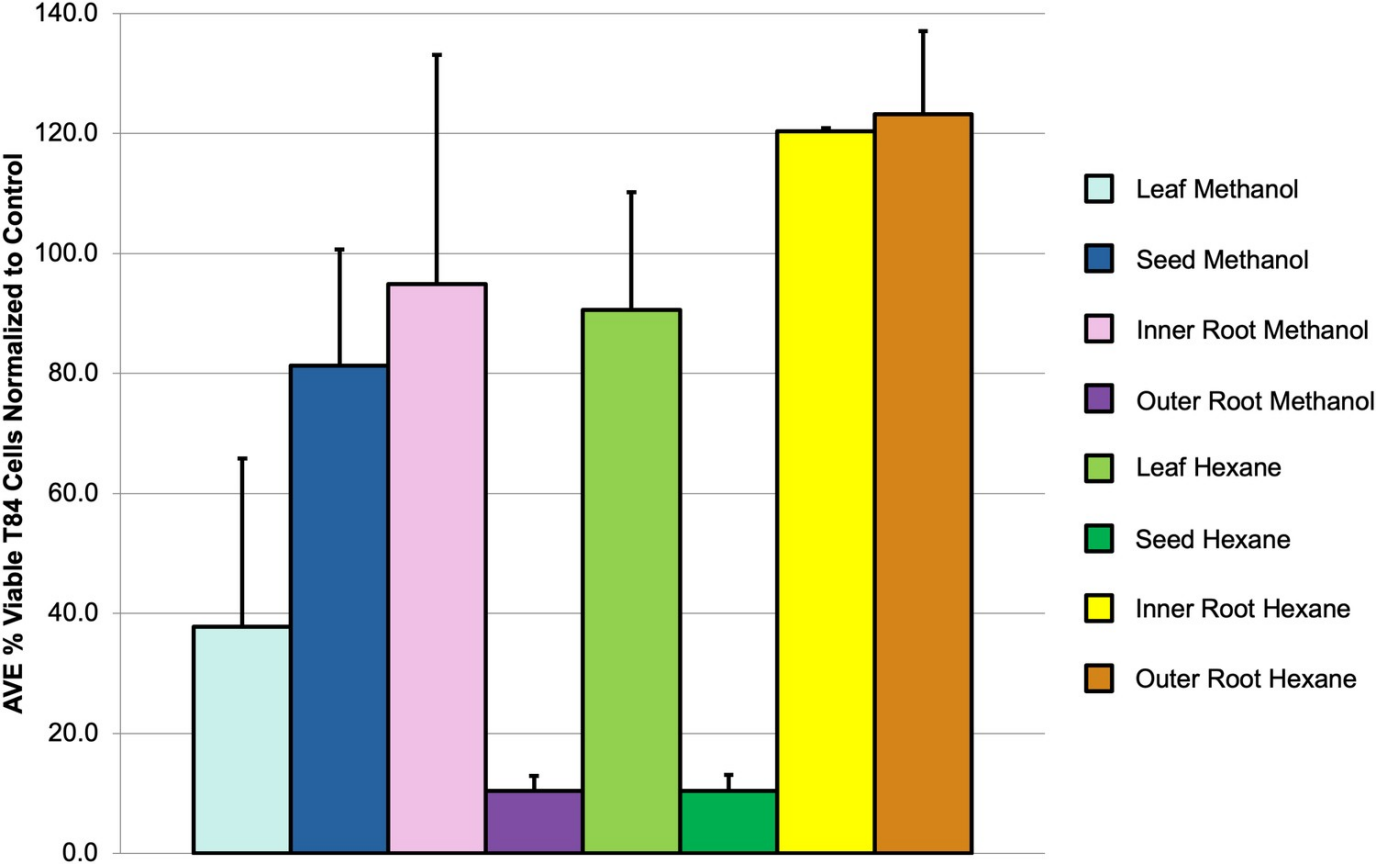
Viability assay of cancer cells. T84 human colon cancer cells were treated with 1 mg of either methanol or hexane extract for leaf, seed, inner root or outer root for 1 h. The MTT colorimetric assay was then used to determine cell metabolic activity after treatment with extracts. The mean percentage of viable cells normalized to the control (solvent alone) for at least three biological replicates is shown with associated SEM.

In addition to antimicrobial activity, the *A*. *mexicana* plant has been reported to possess cytotoxic activity against various human cancer cell lines. For example, the leaf methanol extract was found to possess cytotoxic activity against three human cancer lines, AGS, HT-29 and MDA-MB-435S [26], and both leaf and stem extracts exhibited inhibitory effects against A549, SiHa and KB immortalized cell lines [15]. Moreover, several alkaloids isolated from the whole plant have been shown to inhibit growth of human nasopharyngeal carcinoma (HONE-1) and human gastric cancer (NUGC) cell lines [27]. Colorectal cancer is reported to be the third leading cause of cancer-related deaths in the United States [40]. In this study, *A*. *mexicana* extracts (**Fig 2**) were tested against T84 human colon cancer cells for the first time. As can been seen in **Fig 4**, the outer root methanol and seed hexane extracts were found to have the greatest inhibitory activity against these cells. Interestingly, cells treated with inner and outer root hexane extracts survived better than cells that were treated with hexane alone, with mean viability percentages greater than 100% after being normalized to the control (solvent alone). This suggests that the compounds in these extracts could potentially be promoting cell growth.

**Fig 5:**
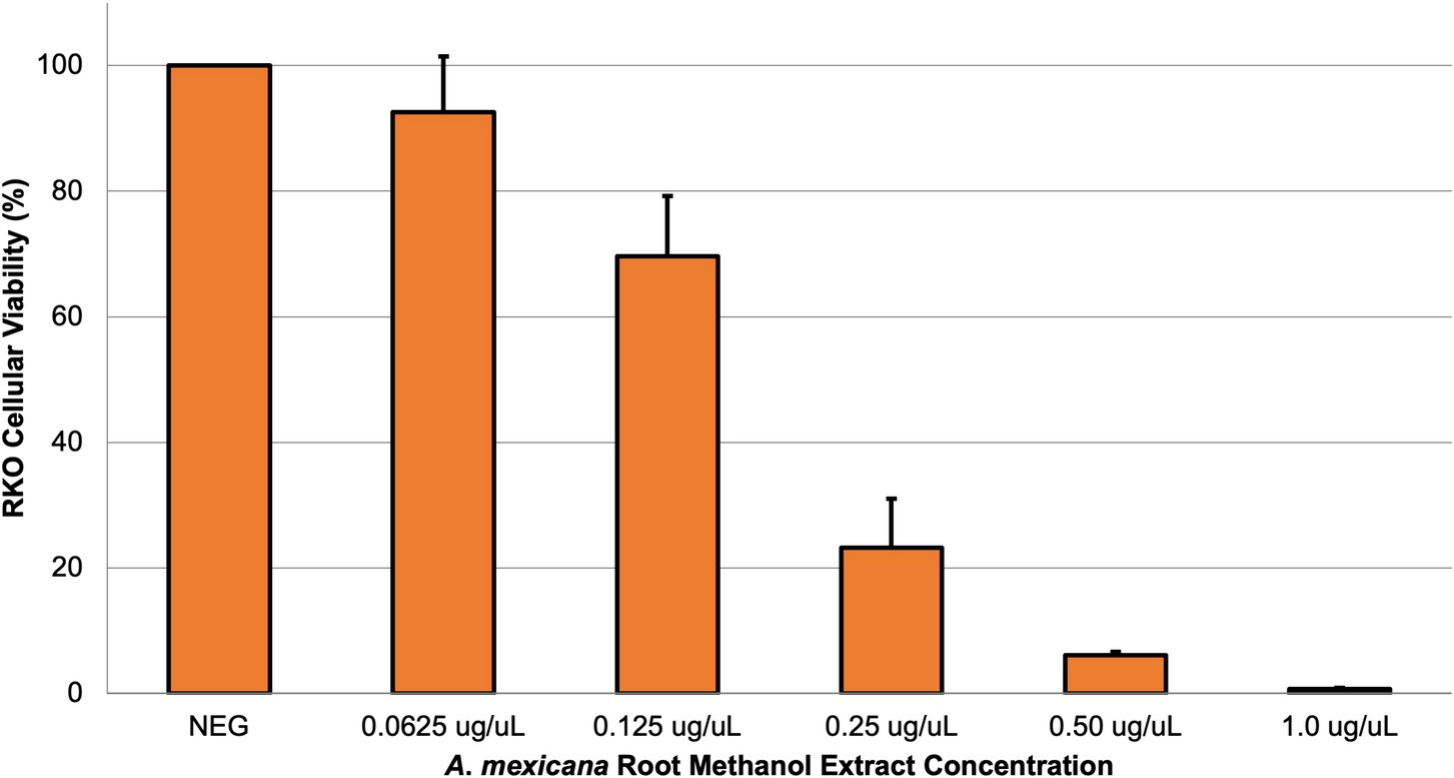
Viability assay of root methanol extract against cancer cells. Human colon cancer RKO cells were treated with 0.0625 μg/μL, 0.125 μg/μL, 0.25 μg/μL, 0.50 μg/μL or 1.0 μg/μL dehydrated root methanol extract or no treatment (evaporated methanol alone, labelled as ‘NEG’) and assessed using the MTT assay for cell viability after 72 h. The mean percentage of viable cells for three biological replicates is shown with associated SEM.

In an effort to begin elucidating how the root methanol extract may be affecting colon cancer cells, changes in the colon cancer oncogene, c-MYC [41], and tumor suppressor gene, APC [42], were evaluated after extract treatment. To determine a non-cytotoxic root methanol concentration, RKO cells were treated with 0.0625 μg/μL, 0.125 μg/μL, 0.25 μg/μL, 0.50 μg/μL or 1.0 μg/μL dehydrated root methanol extract or no treatment (evaporated methanol alone) and assessed for cell viability after 72 h (**Fig 5**).

**Fig 6:**
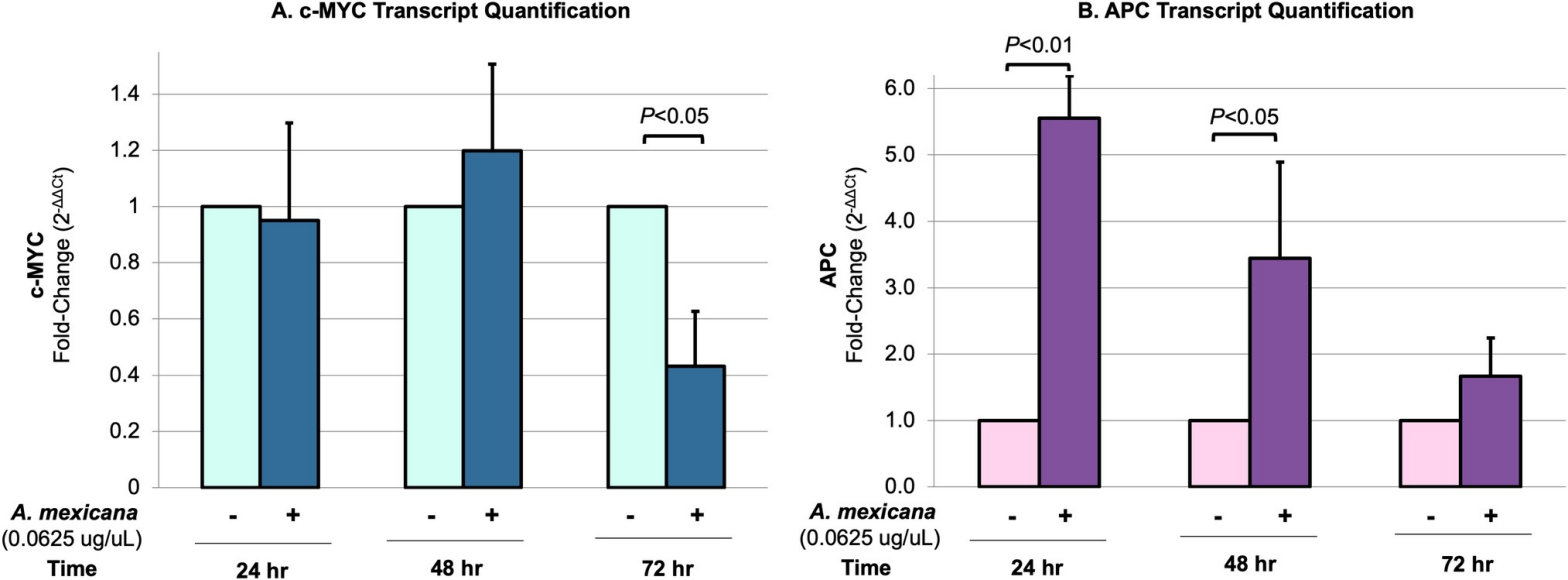
Effect of root methanol extract on c-MYC and APC transcript levels. Human colon cancer RKO cells were treated with or without 0.0625 ug/uL dehydrated *A*. *mexicana* root methanol extract for 24, 48 or 72 h. RNA was then extracted, converted to cDNA and subsequently used in qPCR to quantify the transcript levels of the colon cancer oncogene c-MYC (panel A) or the tumor suppressor gene APC (panel B). The mean transcript level for three biological replicates is shown here with associated SEM and normalized to mRNA levels of the housekeeping gene Actin. The transcript level in the negative control (no treatment) for each condition was set to 1.0. Statistical significance among transcript means was determined through a one-way analysis of variance (ANOVA), with significance set at *P* ≤ 0.05.

Based on this result (**Fig 5**), RKO cells were treated with or without 0.0625 ug/uL root methanol extract, and RNA was harvested 24, 48 and 74 h after treatment. Isolated RNA was then converted to cDNA, which was used in qPCR reactions to determine changes in the transcript levels of the colon cancer oncogene c-MYC or the tumor suppressor gene APC (**Fig 6**). c-MYC mRNA levels were only found to be significantly altered (decreased) after 72 h of *A*. *mexicana* root extract treatment (24h: 5% decrease, *P*>0.05; 48h: 20% increase, *P*>0.05; 72h: 57% decrease, *P*<0.05) (**Fig 6A**). In contrast, APC transcript levels were generally found to increase after treatment (24h: 5.6-fold change, *P*<0.01; 48h: 3.4-fold change, *P*<0.05; 72h: 1.7-fold change, *P*>0.05) (**Fig 6B**). High levels of c-MYC are associated with different types of cancer; this oncogene can regulate aberrant cell proliferation, apoptosis, genomic instability, cell immortalization and chemotherapeutic resistance [43]. However, about 70% of sporadic colon cancer is initiated by biallelic inactivation of the APC gene, which causes aberrant activation of WNT/β-catenin signaling [44]. Although future work is warranted to correlate c-MYC and APC transcript trends with protein levels in colon cancer cells, these preliminary results indicate that *A*. *mexicana* root methanol extract may possess compounds that modulate molecular signaling pathways in colon cancer cells.

As the root methanol extract demonstrated both antibiotic (**Fig 2**) and cytotoxic/potential anticancer (**Figs 4–6**) activity, it was chosen first for separation by chromatography and further chemical analysis, guided by bio-assay results of the antimicrobial disc-diffusion assay. However, to ensure that the antibiotic activity was not due to some compound in the surrounding environment, soil directly from the site of harvest was extracted in methanol via maceration and tested for antimicrobial activity (**S2 Fig**). The soil alone was not found to possess any inhibitory effects against the bacteria *S*. *aureus*.

**Fig 7:**
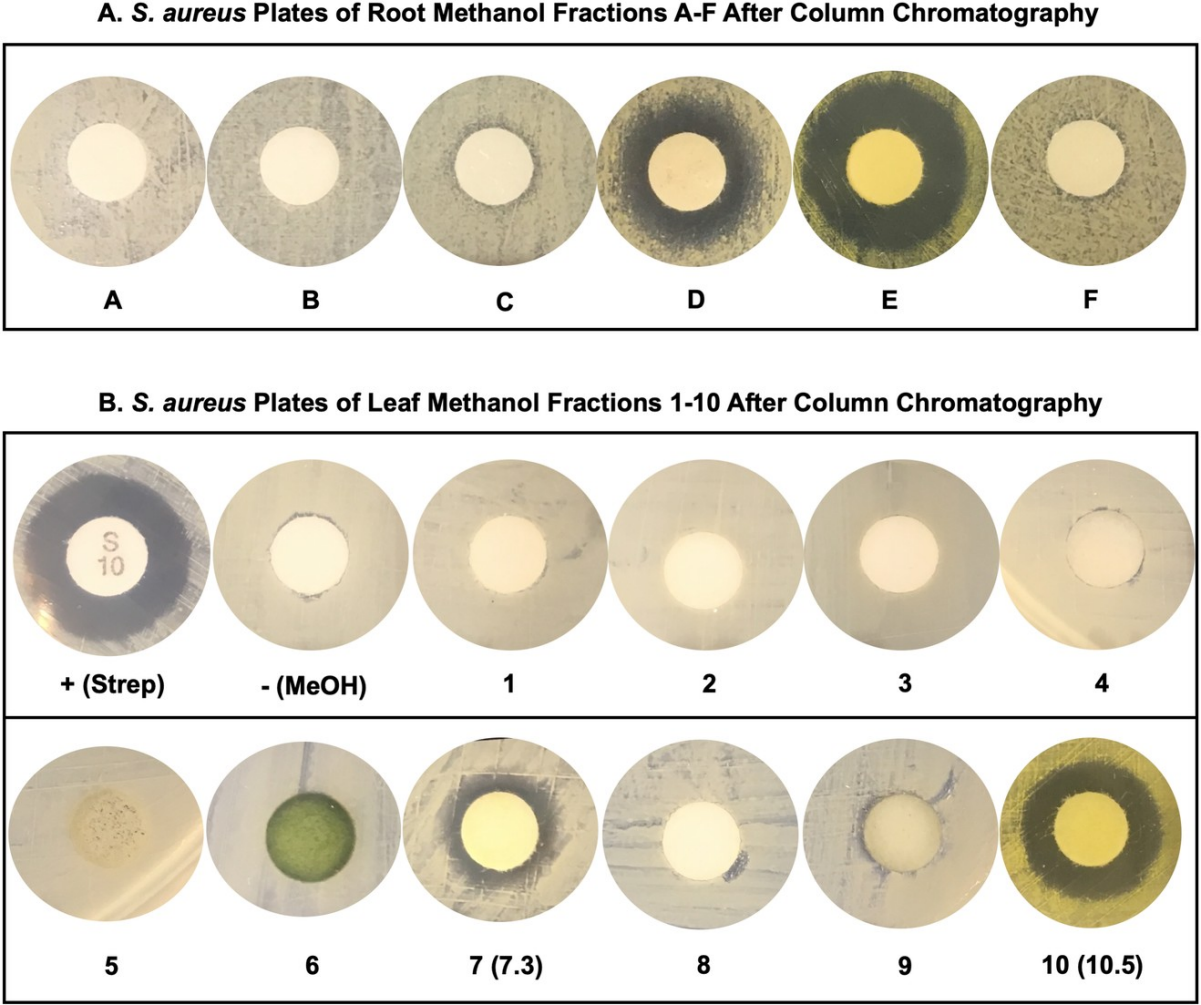
Root and leaf column chromatography *S*. *aureus* plates. Based on the order of column elution, the root methanol fractions (panel A) were arbitrarily labeled A-F, and the leaf methanol fractions (panel B) were arbitrarily labeled 1–10. After multiple separations by column chromatography, these fractions were tested for antimicrobial activity against *S*. *aureus*, with streptomycin and vancomycin used as positive controls and methanol alone as the negative control. (Several representative full plates can be seen in **S4 Fig**) The fractions with strongest antimicrobial activity (root methanol D and E and leaf methanol 7.3 and 10.5) were then evaluated for purity (**S3 Fig**) and used for chemical characterization (**Table 1**).

Using automated column chromatography techniques (see *Methods*), the crude root methanol extract was separated into its individual components, with each sub-fraction arbitrarily labeled A-thru-F. Root sub-fractions ‘D’ (Silica TLC; 9:1 DCM:MeOH; R_f_ = 0.52) and ‘E’ (R_f_ = 0.16) (**S3A Fig**) appear most responsible for the antimicrobial effects (**Fig 7A**). Sub-fractions ‘D’ and ‘E’ were, therefore, submitted to further chromatographic purifications prior to downstream analysis.

Chromatographic separation of the crude leaf methanol extracts was also performed (**S3B Fig**). Fractions ‘7’ and ‘10’ from the leaf extract showed the most activity, and each was further sub-fractionated to 7.1–7.7 and 10.1–10.5, with ‘7.3’ and ‘10.5’ showing the highest antimicrobial activity (**Fig 7B**). Initial analysis of ‘7.3’ revealed it remained a complex mixture and required additional steps before undergoing thorough analysis (as discussed later). Meanwhile, leaf fraction ‘10.5’ showed the same fluorescent properties and TLC R_f_ value seen in fraction ‘E’ from the root.

In an effort to identify the structures of the active components found in the root-D, root-E, and leaf-10.5 sub-fractions, these were analyzed by ultra-performance liquid chromatography coupled with mass spectrometry to determine the masses of their components (**Table 1**). Several representative samples of root-D, root-E, and leaf-10.5 fractions from multiple extracts were analyzed to ensure uniformity across chromatographic separations. The active component of ‘D’ was found to have a mass of 348amu, and this sample was further analyzed by high-resolution mass spectrometry to provide a predicted chemical formula. Similarly, component ‘E’ was found to possess a compound of 336amu, and this was also analyzed by high-resolution mass spectrometry for a predicted formula. Some fractions of ‘E,’ which were found to be of lesser purity, also showed an impurity ion of 370amu. This mass difference was most consistent with the parent 336 mass +H_2_O+CH_3_+H, based on high-resolution mass spectrometry. Analysis of ‘10.5’ from the leaf likewise revealed overlap with fraction ‘E’ from the root. This, coupled with the similar TLC data, strongly suggests the primary antimicrobial component within the root is also distributed in the leaves of the *A*. *mexicana* plant. It was noteworthy that no evidence from TLC or LCMS suggested that the root-D component was present in the original leaf extract or fractions collected via chromatography.

**Table 1:** Summary of mass spectrometry results from active sub-fractions of plant extracts.

Fraction	R_f_^a^	High-Resolution Mass	Predicted Chemical Formula^b^
Root-D	0.52	348.1250	C_21_H_18_NO_4_
Root-E	0.16	336.1256	C_20_H_18_NO_4_
		370.1666 (minor impurity)^c^	C_21_H_24_NO_5_
Leaf-10.5	0.15	336.1255	C_20_H_18_NO_4_
		354.1339 (impurity)	C_20_H_20_NO_5_

a: silica TLC (9:1 DCM:MeOH)

b: as determined through high-resolution mass spectrometry

c: this impurity was not detected in all E samples

The heat stability of the antimicrobial compounds in each of the purified root and leaf fractions was also evaluated, and after treatment at 100°C for 10 min, no major changes were observed in R_f_ values, fluorescent properties or antibacterial activity (**S5 Fig**).

**Fig 8:**
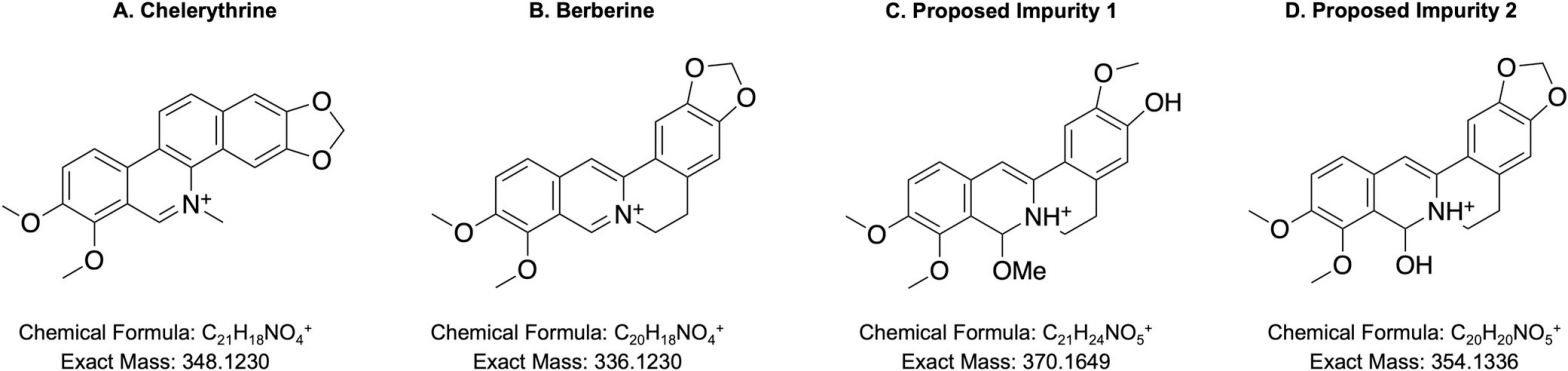
Expected structures of key active fractions of the root and leaf methanol extract. A) Structure of chelerythrine, consistent with root fraction ‘D’. B) Structure of berberine, consistent with root fraction ‘E’ and leaf fraction ‘10.5’. C) Proposed structure of the impurity observed in root fraction ‘E’. D) Proposed structure of the impurity observed in leaf fraction ‘10’.

Components ‘D’, ‘E’ and ‘10.5’ were then compared to a list of all known compounds that have been identified as *A*. *mexicana* chemical constituents [31]. Chelerythrine had a mass consistent with root fraction ‘D’, while berberine was consistent with that of root ‘E’ and leaf ‘10.5’ (**Fig 8**). Both chelerythrine and berberine are N-containing alkaloids with reported antibacterial, anticancer, anti-inflammatory activities (reviewed in [45]). Chelerythrine acts as an antibiotic through destruction of the bacterial cell wall as well as by degradation of the cell membrane and inhibition of protein biosynthesis [46], while the mechanism of berberine is believed to occur through damaging the bacterial membrane and inhibiting synthesis of protein and DNA [47]. As previously mentioned, there was no evidence of component ‘D’ in the leaves, suggesting that while the roots contain both of these phytocompounds, chelerythrine is absent in the leaves. These results are consistent with the data shown in **Fig 2**, where the root extracts exhibit stronger effects against the Gram-positive bacteria, which possess a much thicker peptidoglycan cell wall than Gram-negative bacteria.

**Fig 9:**
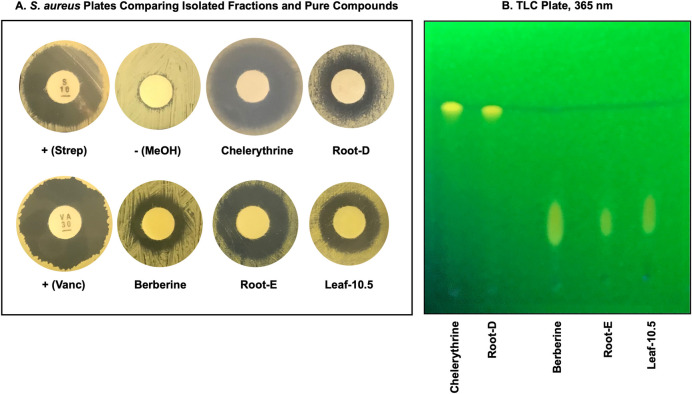
Comparison of known compounds to antimicrobial root and leaf methanol fractions. Commercially available chelerythrine was compared to root extract fraction ‘D,’ while berberine was compared to and root fraction ‘E’ and leaf fraction ‘10.5’. Antimicrobial effects against *S*. *aureus* are shown in Panel A, with streptomycin and vancomycin used as positive controls and methanol alone as a negative control. A representative TLC plate (under 365 nm exposure) is shown in Panel B.

To assess the validity of these potential matches, authentic samples of chelerythrine and berberine were purchased (Sigma-Aldrich, St. Louis, MO, USA) and compared for antimicrobial activity on *S*. *aureus* plates and by TLC to determine whether these compounds were consistent with the bioactive components from the extractions (**Fig 9**). Both antimicrobial activity and thin-layer chromatography strongly suggest these compounds represent a positive match. Data from ultra-performance liquid chromatography coupled with mass spectrometry was also identical between root-D and chelerythrine, as well as root-E/Leaf-10.5 and berberine.

As a final confirmation of these matches, nuclear magnetic resonance (NMR) analyses were performed on samples of root-D, root-E, and leaf-10.5, which had been rigorously purified from pooled large-batch extracts (**S6 thru S10 Figs**). The ^1^H-NMR spectra showed that root-D was identical to chelerythrine [48], while root-E and leaf-10.5 were identical to berberine [49]. ^13^C-NMR data were also able to be collected for the two compounds from the root, which was consistent with literature reports [48, 49]. These NMR results, coupled with the matching data from TLC and ultra-performance liquid chromatography coupled with mass spectrometry, confirms these structural matches.

Based on the strong evidence that fraction ‘E’ is berberine, this helps to identify the two minor impurities detected. A common isolation-artifact of berberine is a water-adduct, whereby solvent adds to the reactive iminium [50]. This is consistent with the 354amu impurity found in ‘10.5’ of the leaf (**Fig 8D**). Following this, it is likely the 370amu impurity is a result of the bioactive component undergoing a reductive cleavage of the methylene to initially give jatrorrhizine, followed by methanol addition to the reactive iminium group (**Fig 8C**). The methanol adduct is likely formed during the extraction phase, and it is noteworthy that no evidence was detected of the water-adduct of this berberine variant, or jatrorrhizine alone.

As previously stated, the leaf sub-fraction ‘7.3’ was initially found to consist of a complex mixture. LCMS analysis of this fraction showed masses that did not align with the antimicrobial components found in the root extract. Further attempts to purify and identify the antimicrobial compounds in leaf subfraction ‘7.3’ have been made. Given the common impurities encountered in the berberine-containing samples, it was reasoned that the impurity in ‘7.3’ may also stem from similar solvent-adducts. There is precedent that the free-base of such alkaloids can convert to bimolecular aminal-ethers, and therefore they may be more stable in acidic environment [51]. A noteworthy result was found when treating the crude leaf methanol extract with HCl, followed by extraction with dichloromethane after neutralizing the aqueous phase, as TLC analysis of this DCM extract revealed new and/or enhanced components when compared to the original leaf extract (**S11A Fig**). In place of the dark spot, which initially corresponded to ‘7.3’, was a bright fluorescent orange spot (**S11A Fig**). Isolation of the new orange fraction by Prep-TLC and analysis via mass spectrometry revealed the presence of sanguinarine (332.0952amu) and chelerythrine (348.1227amu). As previously indicated, there was no previous detection of chelerythrine in the original leaf extract or its sub-fractions, suggesting the initial extract may contain inactive proto-alkaloid derivatives, which are only converted to their active form upon treatment with acid. This is supported as ‘7.3’ has a nearly identical R_f_ compared to chelerythrine and sanguinarine but lacks the characteristic fluorescent properties (**S11B Fig**). Treatment of the separated ‘7.3’ fraction with methanolic HCl resulted in this same modification to the TLC results. The activity of fraction ‘7.3’ may stem from an acid-catalyzed transformation to sanguinarine and chelerythrine during transport or uptake into the bacterial cell.

## Conclusions

In conclusion, this work serves as a comprehensive characterization of the cytotoxic activities of various parts (using several developmental states) of *A*. *mexicana* against multiple bacterial, fungal and human colon cancer cells. It also sheds light upon the allocation of several major antibiotic compounds (chelerythrine and berberine) in the roots and leaves of this plant. Additionally, upon treatment with *A*. *mexicana* root methanol extract, the previously unexplored RKO colon cancer cells were found to downregulate the c-MYC oncogene and upregulate the tumor suppressor APC gene, indicating the potential of this plant in colon cancer therapeutics. Such findings may serve as a starting point for uncovering more of the compounds that give *A*. *mexicana* its many unique medicinal properties.

## Supporting information

S1 Fig Traditional vs. Soxhlet extraction comparison. 2 g of plant material was extracted in methanol using either the traditional (as in ‘Materials and methods’) or Soxhlet extraction procedure. 1 mg of each root or leaf methanol extract was plated against *S*. *aureus*, for three total replicates per extraction type (one representative root methanol plate is shown in panel A). For all bacterial plates, streptomycin and vancomycin were used as positive controls, and methanol alone was used as a negative control. Total extraction yields were also compared between extraction methods for both root and leaf methanol (displayed in panel B).
(TIF)

S2 Fig Soil methanol extraction. 2 g of soil per replicate from the *A*. *mexicana* plant harvest site was used to perform methanol extractions following the same extraction protocol outlined in ‘Materials and methods’. 1 mg of each replicate was then plated against *S*. *aureus*. No zones of inhibition were observed for any of the three soil extraction replicates. Streptomycin, vancomycin and 1 mg unpurified root methanol extract were used as positive controls, and methanol alone was used as a negative control.
(TIF)

S3 Fig Separation of antimicrobial compounds from root and leaf methanol extracts. Normal-phase column chromatography was performed to separate root and leaf methanol extract compounds. The fractions with the strongest antimicrobial activity against *S*. *aureus* (root D and E, and leaf 7.3 and 10.5, as shown in **Fig 7**) were evaluated for purity using thin layer chromatography. Representative TLC plates are shown above for the root compounds (panel A) and for the leaf compounds (panel B), where ‘Crude RM’ is the root methanol extract before separation and ‘Crude LM’ is the leaf methanol extract before separation.
(TIF)

S4 Fig Column chromatography *S. aureus* plates. Several representative full, uncropped plates (referred to in **Fig 7**) of separated root (panel A) and leaf (panel B) methanol fractions tested for antimicrobial activity against *S*. *aureus*, with streptomycin, vancomycin and 1 mg unpurified root or leaf methanol extract as positive controls and methanol alone as the negative control.
(TIF)

S5 Fig Heat stability of purified compounds. Selected root (D and E, panel A) and leaf (7.3, 9.6 and 10.5, panel B) methanol fractions were treated at 100°C for 10 min and subsequently tested for heat stability by comparison to untreated controls on TLC (upper panel) and *S*. *aureus* (lower panel) plates, where ‘NT’ refers to no treatment. On the antimicrobial plates, vancomycin and 1 mg of unpurified extracts were used as positive controls, and methanol alone was used as a negative control.
(TIF)

S6 Fig ^1^H-NMR spectrum of root-D. This spectrum matches that of an authentic sample of chelerythrine. The signals at 5.3ppm and 3.5ppm represent dichloromethane and methanol, respectively, which are residual solvent peaks from the mobile phase during purification.
(TIF)

S7 Fig ^13^C-NMR spectrum of root-D. This spectrum matches that of an authentic sample of chelerythrine. Due to issues with solubility at concentrations needed for ^13^C-NMR, this spectrum was taken using deuterated methanol as the solvent.
(TIF)

S8 Fig ^1^H-NMR spectrum of root-E. This spectrum matches that of an authentic sample of berberine. The signals at 5.3ppm and 3.5ppm represent dichloromethane and methanol, respectively, which are residual solvent peaks from the mobile phase during purification.
(TIF)

S9 Fig ^13^C-NMR spectrum of root-E. This spectrum matches that of an authentic sample of berberine. The signal at 50ppm represents residual methanol.
(TIF)

S10 Fig ^1^H-NMR spectrum of leaf-10.5. This spectrum matches that of an authentic sample of berberine. The signals at 5.3ppm and 3.5ppm represent dichloromethane and methanol, respectively, which are residual solvent peaks from the mobile phase during purification. See **S8 Fig** for structural alignment with Berberine.
(TIF)

S11 Fig Effect of HCl treatment on crude leaf methanol extract. A) Appearance of new/enhanced components on TLC plate after treatment with HCl. B) TLC comparison of R_f_ values of ‘7.3’, the new components post-HCl treatment, sanguinarine and chelerythrine.
(TIF)
