# Correction: Characterizing the cytotoxic effects and several antimicrobial phytocompounds of *Argemone mexicana*

**DOI:** 10.1371/journal.pone.0287803

**Published:** 2023-06-23

**Authors:** Danielle Annette Orozco-Nunnelly, Jeffery Pruet, Clara Patricia Rios-Ibarra, Estefany Lucia Bocangel Gamarra, Theodore Lefeber, Teodora Najdeska

The second author’s first name is spelled incorrectly. The correct name is: Jeffrey Pruet.
