# Supplementary figures and images for: Characterizing the cytotoxic effects and several antimicrobial phytocompounds of Argemone mexicana
Source: PLoS One. 2021 Apr 7;16(4):e0249704. doi: 10.1371/journal.pone.0249704 (PMC8026029; doi:10.1371/journal.pone.0249704)

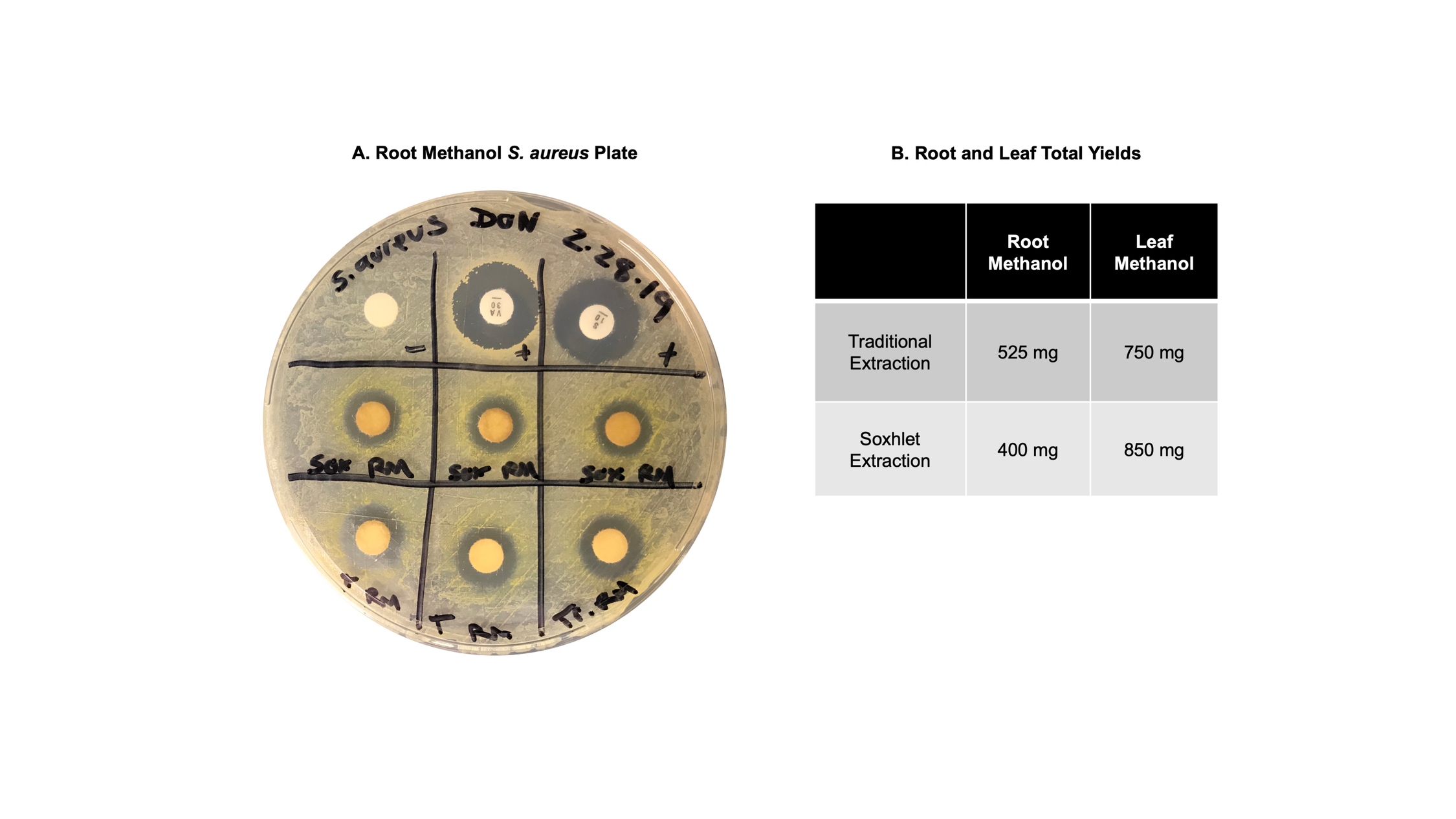

Supplement: S1 Fig — 2 g of plant material was extracted in methanol using either the traditional (as in ‘Materials and methods’) or Soxhlet extraction procedure. 1 mg of each root or leaf methanol extract was plated against S. aureus, for three total replicates per extraction type (one representative root methanol plate is shown in panel A). For all bacterial plates, streptomycin and vancomycin were used as positive controls, and methanol alone was used as a negative control. Total extraction yields were also compared between extraction methods for both root and leaf methanol (displayed in panel B). (TIF) [file pone.0249704.s001.tif]

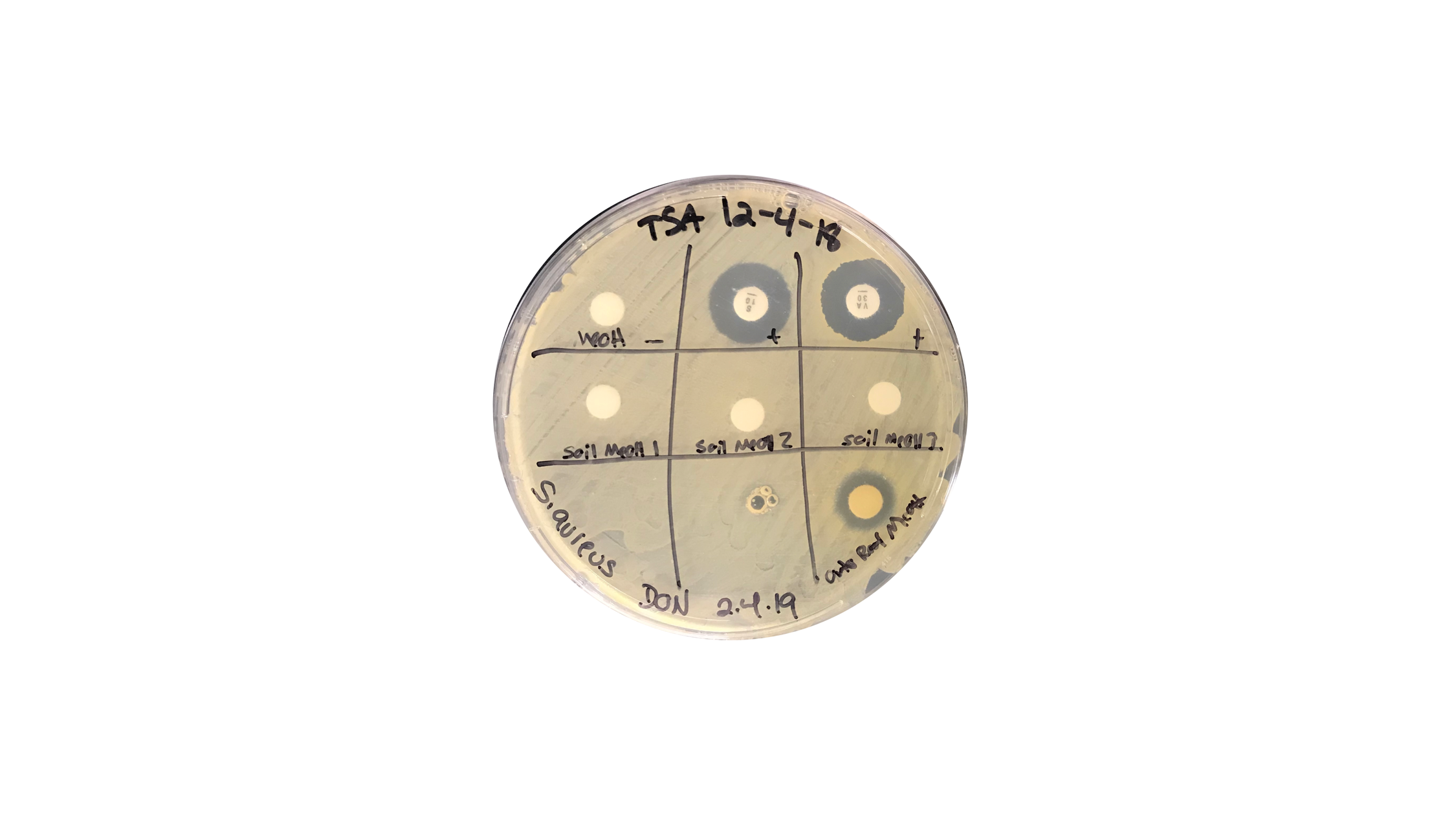

Supplement: S2 Fig — 2 g of soil per replicate from the A. mexicana plant harvest site was used to perform methanol extractions following the same extraction protocol outlined in ‘Materials and methods’. 1 mg of each replicate was then plated against S. aureus. No zones of inhibition were observed for any of the three soil extraction replicates. Streptomycin, vancomycin and 1 mg unpurified root methanol extract were used as positive controls, and methanol alone was used as a negative control. (TIF) [file pone.0249704.s002.tif]

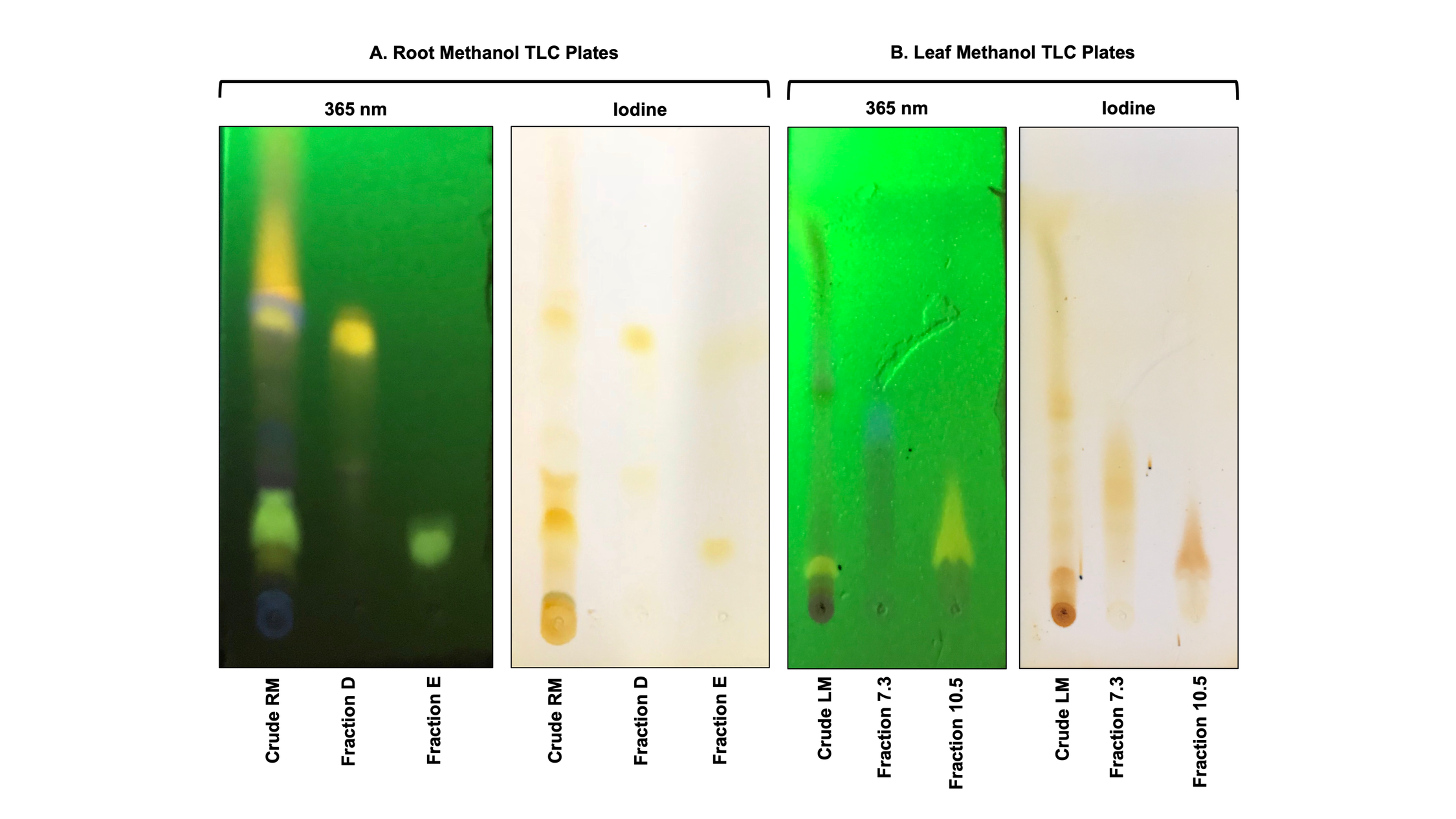

Supplement: S3 Fig — Normal-phase column chromatography was performed to separate root and leaf methanol extract compounds. The fractions with the strongest antimicrobial activity against S. aureus (root D and E, and leaf 7.3 and 10.5, as shown in Fig 7) were evaluated for purity using thin layer chromatography. Representative TLC plates are shown above for the root compounds (panel A) and for the leaf compounds (panel B), where ‘Crude RM’ is the root methanol extract before separation and ‘Crude LM’ is the leaf methanol extract before separation. (TIF) [file pone.0249704.s003.tif]

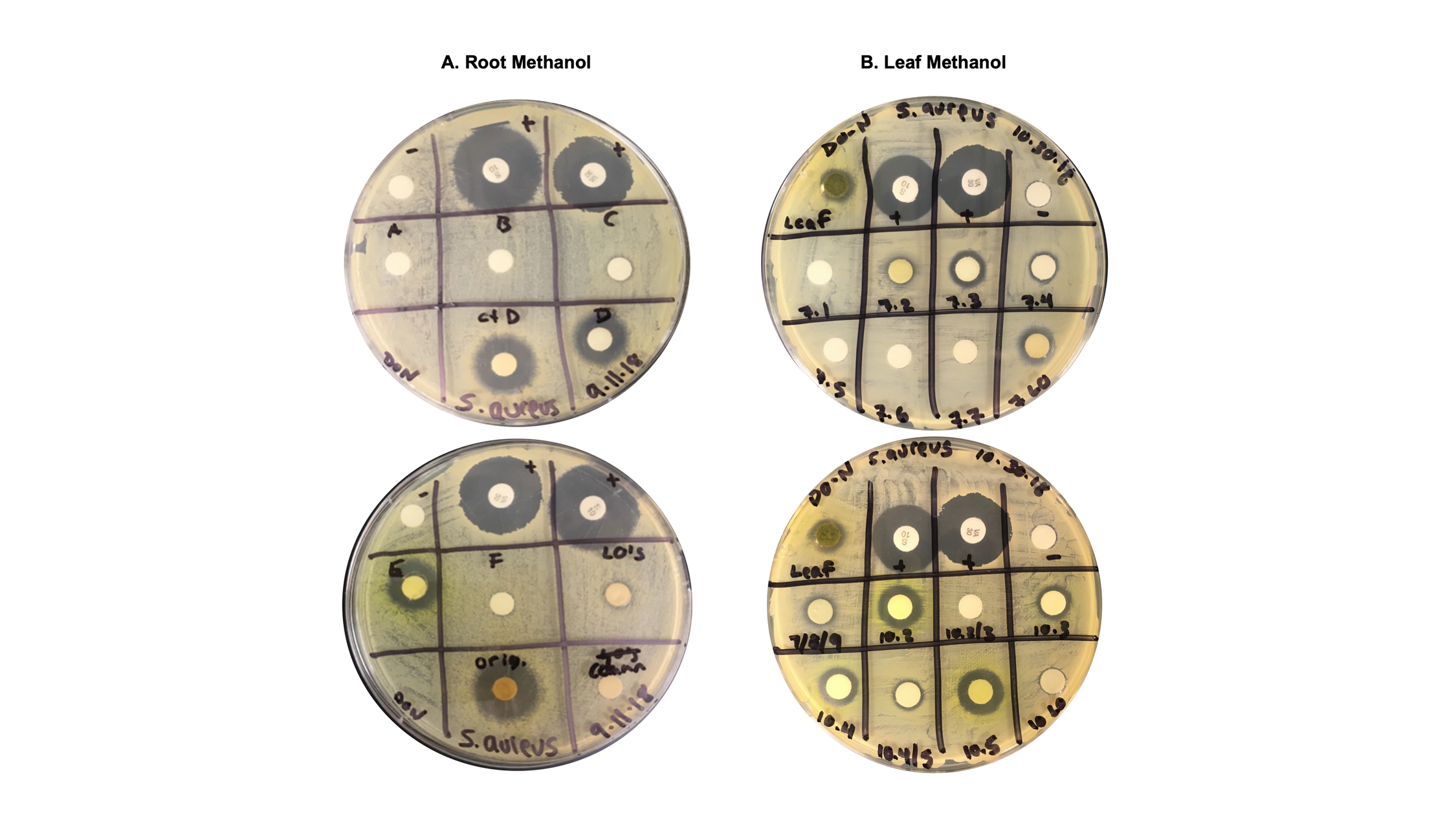

Supplement: S4 Fig — Several representative full, uncropped plates (referred to in Fig 7) of separated root (panel A) and leaf (panel B) methanol fractions tested for antimicrobial activity against S. aureus, with streptomycin, vancomycin and 1 mg unpurified root or leaf methanol extract as positive controls and methanol alone as the negative control. (TIF) [file pone.0249704.s004.tif]

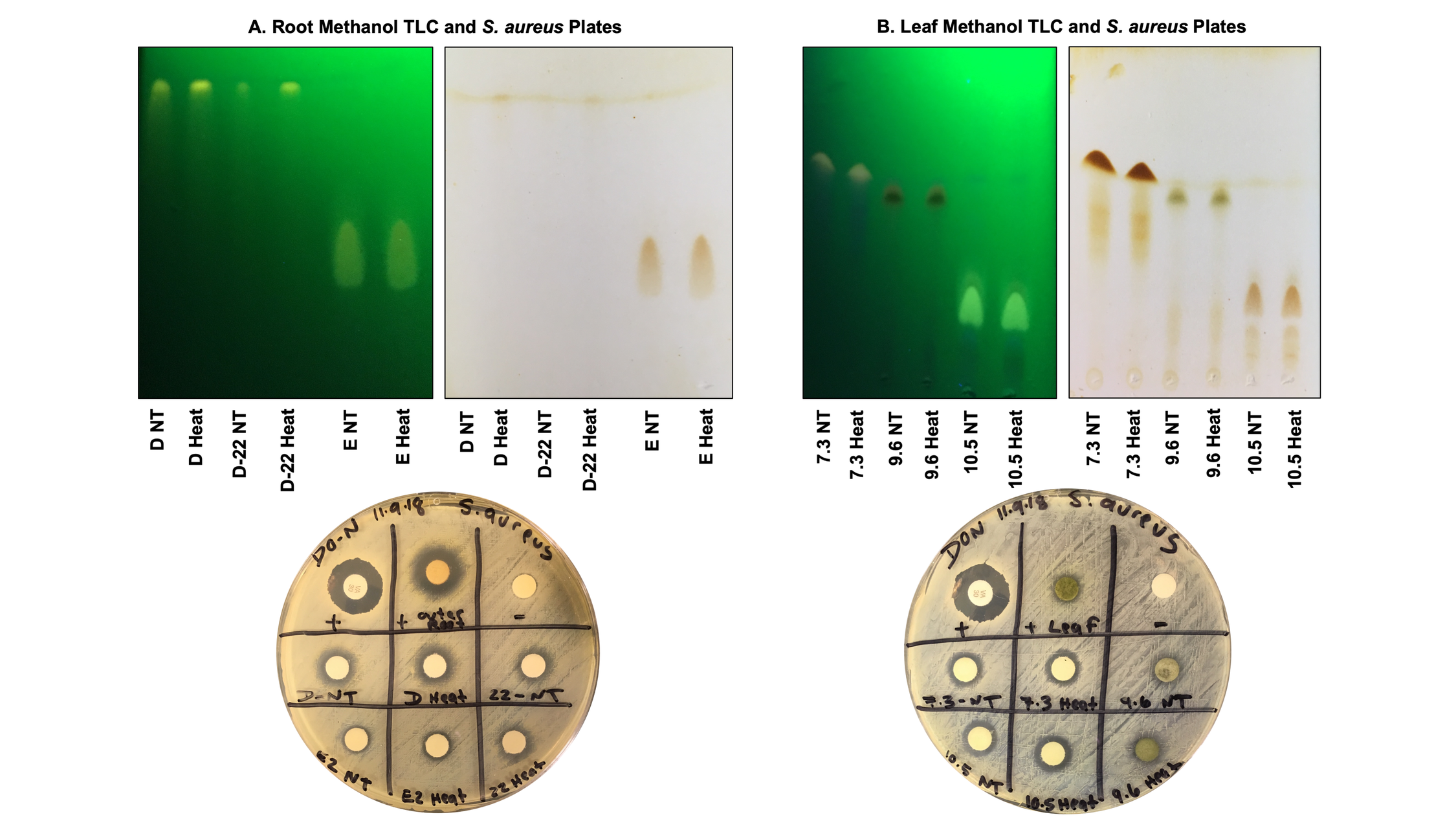

Supplement: S5 Fig — Selected root (D and E, panel A) and leaf (7.3, 9.6 and 10.5, panel B) methanol fractions were treated at 100°C for 10 min and subsequently tested for heat stability by comparison to untreated controls on TLC (upper panel) and S. aureus (lower panel) plates, where ‘NT’ refers to no treatment. On the antimicrobial plates, vancomycin and 1 mg of unpurified extracts were used as positive controls, and methanol alone was used as a negative control. (TIF) [file pone.0249704.s005.tif]

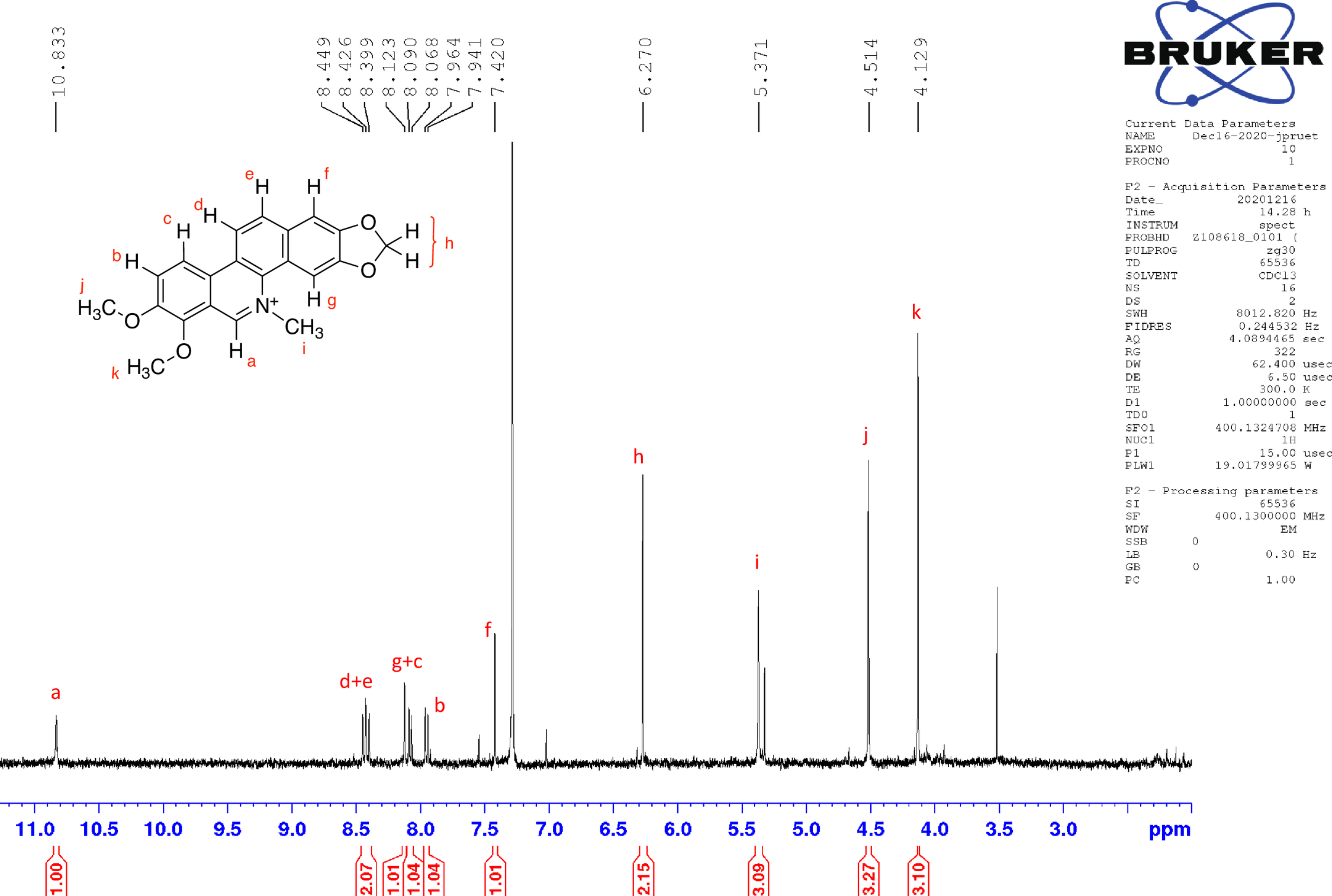

Supplement: S6 Fig — This spectrum matches that of an authentic sample of chelerythrine. The signals at 5.3ppm and 3.5ppm represent dichloromethane and methanol, respectively, which are residual solvent peaks from the mobile phase during purification. (TIF) [file pone.0249704.s006.tif]

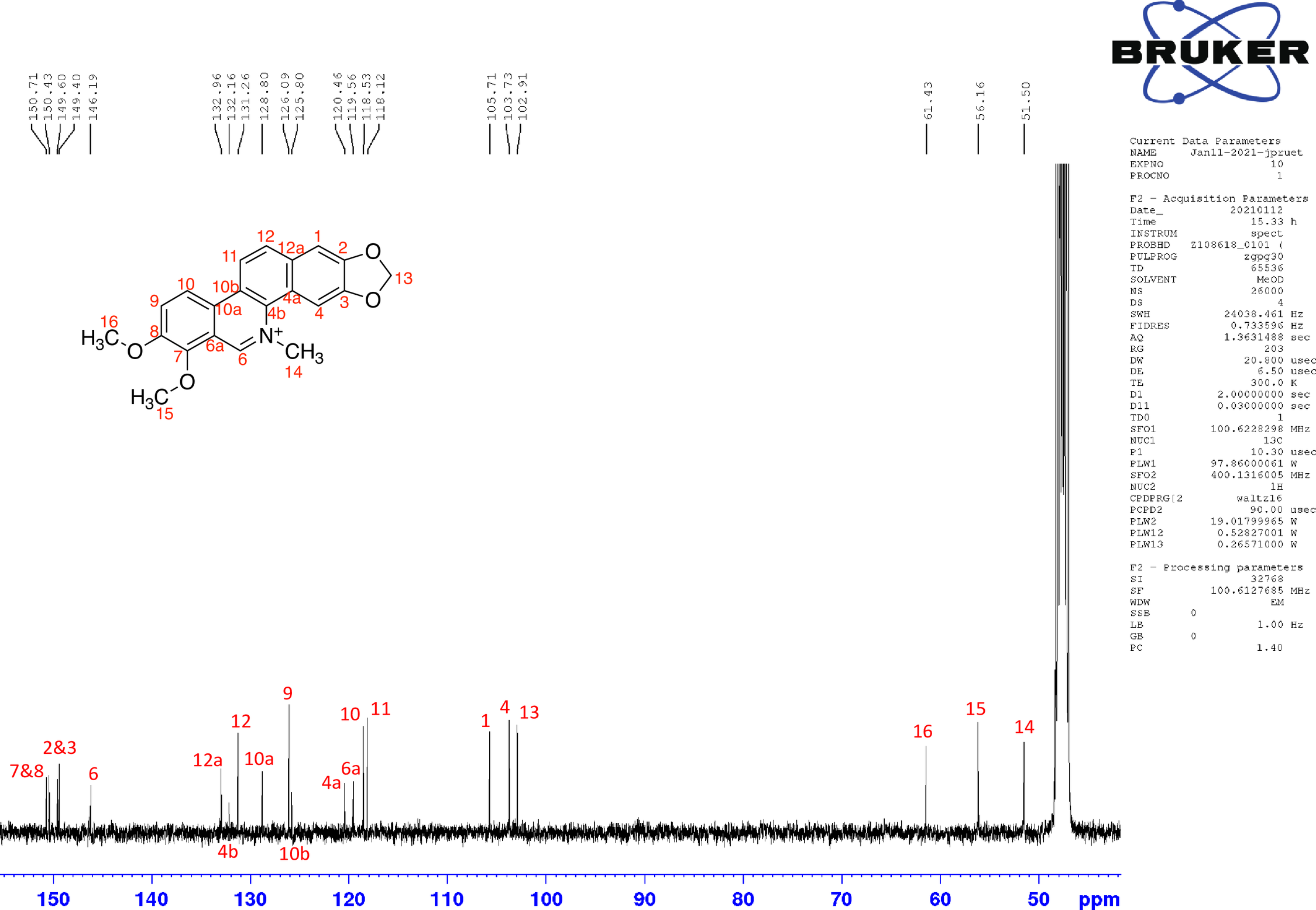

Supplement: S7 Fig — This spectrum matches that of an authentic sample of chelerythrine. Due to issues with solubility at concentrations needed for 13C-NMR, this spectrum was taken using deuterated methanol as the solvent. (TIF) [file pone.0249704.s007.tif]

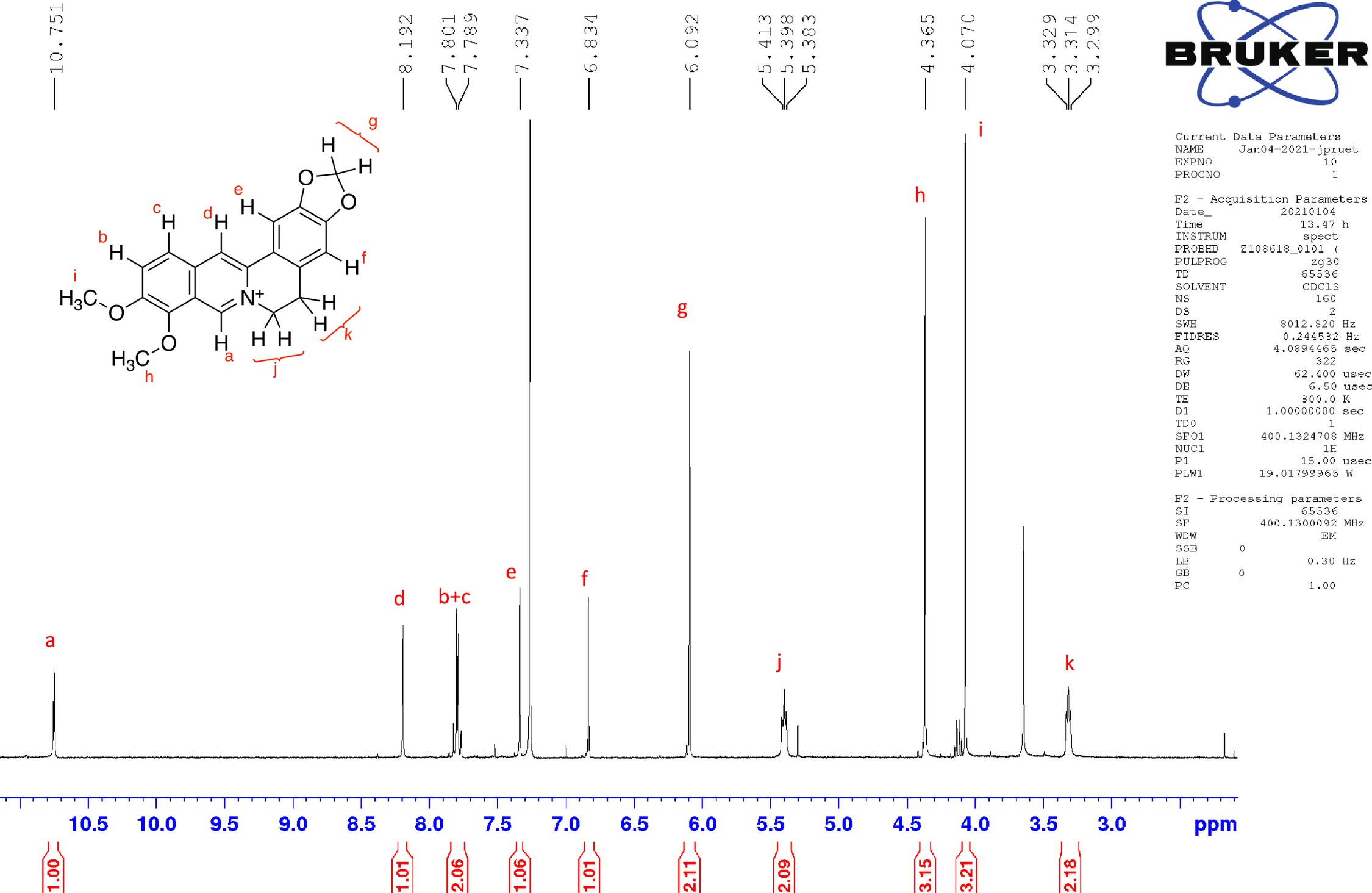

Supplement: S8 Fig — This spectrum matches that of an authentic sample of berberine. The signals at 5.3ppm and 3.5ppm represent dichloromethane and methanol, respectively, which are residual solvent peaks from the mobile phase during purification. (TIF) [file pone.0249704.s008.tif]

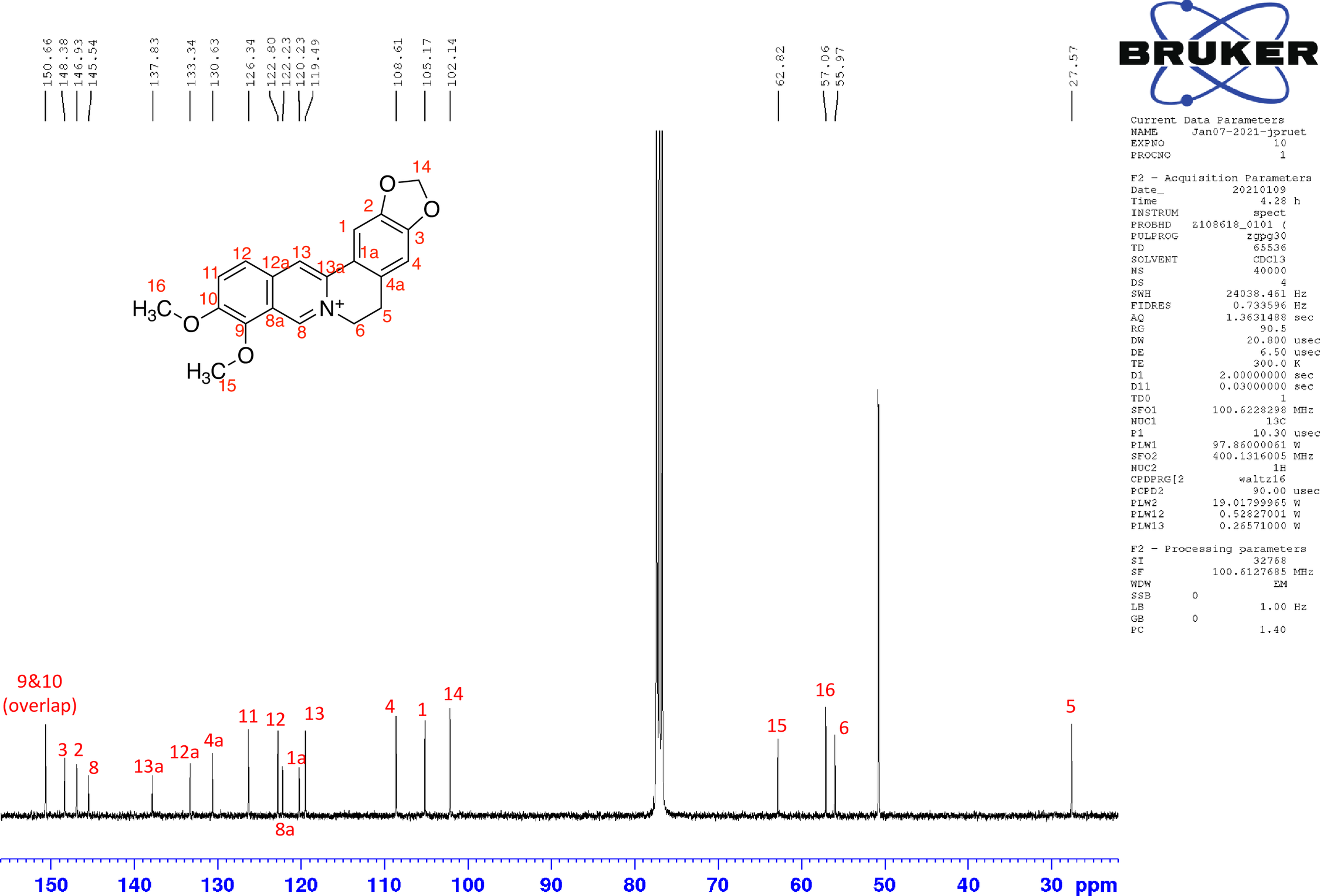

Supplement: S9 Fig — This spectrum matches that of an authentic sample of berberine. The signal at 50ppm represents residual methanol. (TIF) [file pone.0249704.s009.tif]

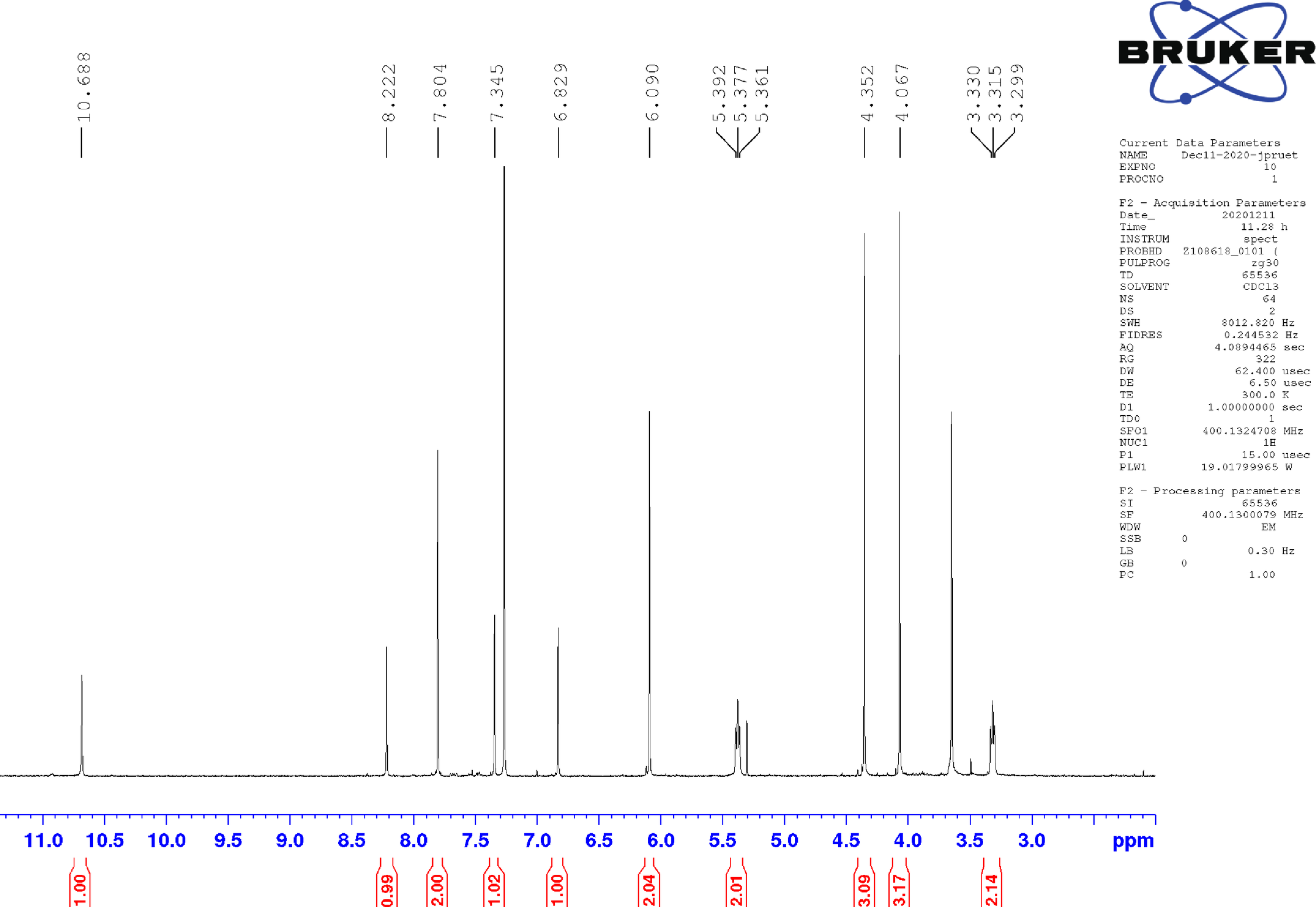

Supplement: S10 Fig — This spectrum matches that of an authentic sample of berberine. The signals at 5.3ppm and 3.5ppm represent dichloromethane and methanol, respectively, which are residual solvent peaks from the mobile phase during purification. See S8 Fig for structural alignment with Berberine. (TIF) [file pone.0249704.s010.tif]

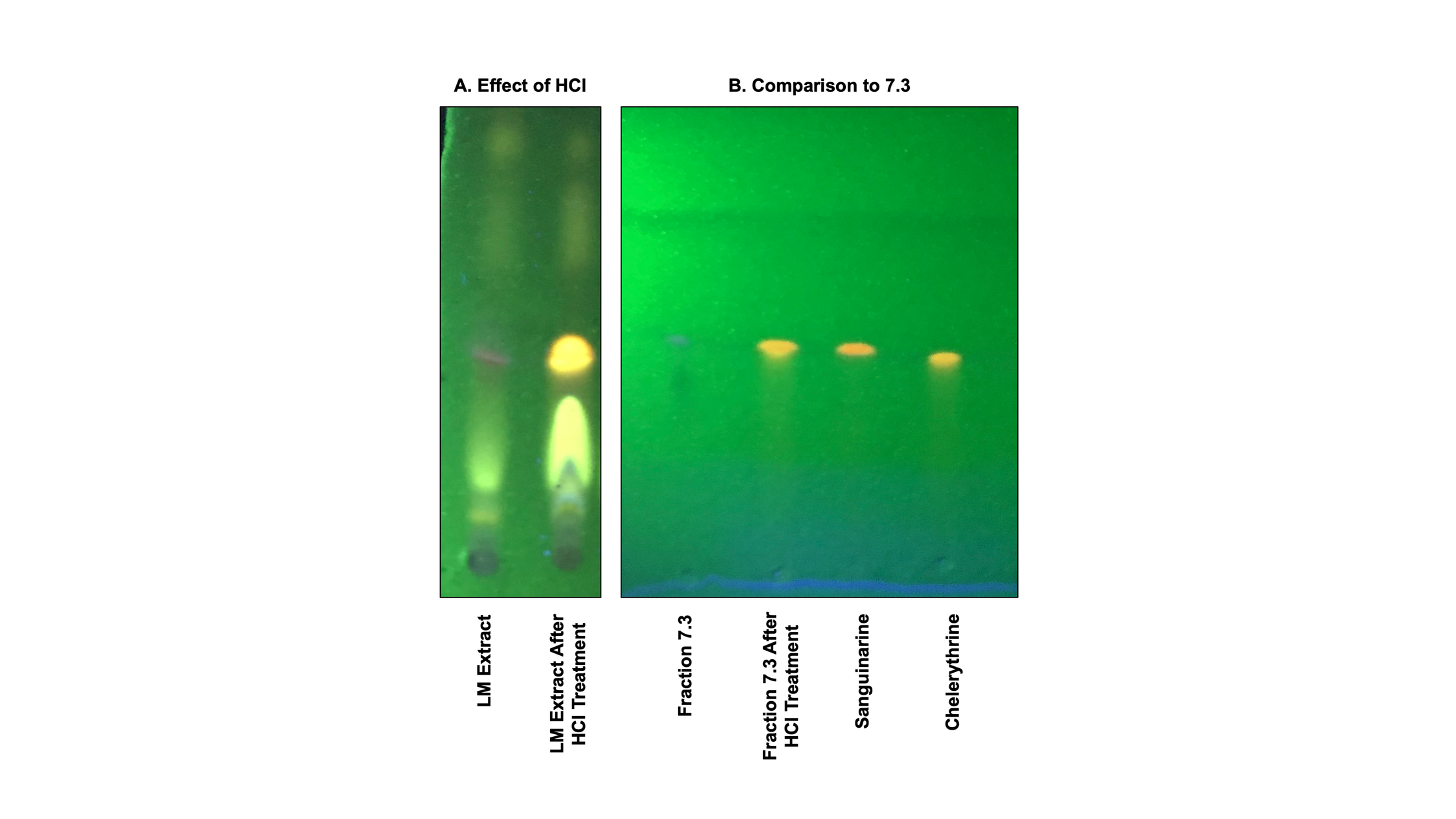

Supplement: S11 Fig — A) Appearance of new/enhanced components on TLC plate after treatment with HCl. B) TLC comparison of Rf values of ‘7.3’, the new components post-HCl treatment, sanguinarine and chelerythrine. (TIF) [file pone.0249704.s011.tif]
